# Triazole–Au(I) complex as chemoselective catalyst in promoting propargyl ester rearrangements

**DOI:** 10.3762/bjoc.7.115

**Published:** 2011-07-25

**Authors:** Dawei Wang, Yanwei Zhang, Rong Cai, Xiaodong Shi

**Affiliations:** 1 Department of Chemistry, West Virginia University, Morgantown, WV 26506, USA

**Keywords:** allene, chemoselectivity, gold catalysis, ligand effect, organometallic

## Abstract

Triazole–Au (TA–Au) catalysts were employed in several transformations involving propargyl ester rearrangement. Good chemoselectivity was observed, which allowed the effective activation of the alkyne without affecting the reactivity of the allene ester intermediates. These results led to the investigation of the preparation of allene ester intermediates with TA–Au catalysts under anhydrous conditions. As expected, the desired 3,3-rearrangement products were obtained in excellent yields (generally >90% yields with 1% loading). Besides the typical ester migrating groups, carbonates and carbamates were also found to be suitable for this transformation, which provided a highly efficient, practical method for the preparation of substituted allenes.

## Introduction

**Scheme 1:**
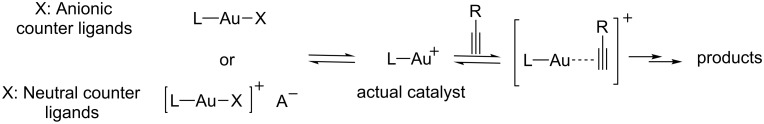
The counter ligands, an important factor in Au(I) catalysis.

The past decade has seen rapid growth in the use of homogeneous gold catalysis for conducting powerful organic transformations [1–9]. Like many other transition metal complexes, the reactivity of gold catalysts greatly depends on the nature of the ligands coordinating with the metal cations [10–15]. Of the two typical oxidation states, Au(I) and Au(III), more studies have been focused on the former cation due to the easier preparation of the catalyst and better pre-catalyst stability. It is currently accepted by the research community that Au(I) complexes adopt one of two coordination sites with 180° linear geometry (Scheme 1) (although some exceptions exist). The actual catalysts involved in alkyne and alkene activation are of the type [L–Au]^+^, with the open coordination site on the opposite side of the ligand (L) for substrate binding [5–6]. The recent success in obtaining the complexes of the alkyne-coordinated [L–Au]^+^, reported by Toste and coworkers, greatly supported this mechanistic model [16]. Generally, the PR_3_ compounds can be applied as the ligand in Au(I) catalysis. The recent development of N-heterocyclic carbene (NHC) derivatives has significantly expanded the choice of ligands by improving the catalyst stability through metal-ligand backbonding [17–20]. To access the active catalyst [L–Au]^+^, stable precursors L–Au–X or [L–Au–X]^+^·A^−^ were typically used. While the ligands (L) are certainly considered critical in gold catalysis, more and more attention have been paid to the evaluation of whether the choice of counter ligand “X” can be used to adjust the overall catalyst reactivity.

**Scheme 2:**
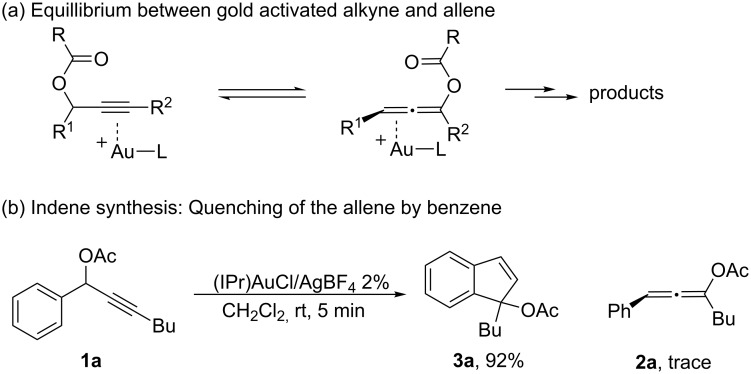
The challenge of the synthesis of allenes through gold activated alkynes.

The propargyl ester rearrangement was considered as one of the most important reaction modes in the Au(I) promoted transformation [21–31]. Recent experimental and computational mechanistic studies revealed the 3,3-rearrangement to form the allene ester intermediate [32–33] as the key step in this transformation (Scheme 2a) [34]. Both experimental and theoretical investigations confirmed the reversibility between allene and propargyl ester due to effective activation of both functional groups by the Au(I) catalysts. As a result, it was extremely challenging to obtain the allene intermediates with good yields. Many strategies have been developed to make the Au(I)-activated allene esters react with other proper substrates, forming interesting new products in a cascade fashion. The indene synthesis (Scheme 2b), reported by Nolan and coworkers, is one good example highlighting the importance of the cascade process [35].

As shown in Scheme 2b, with the [IPr–Au]^+^ catalyst, only trace amount of the allene **2a** was obtained. The major product derived from the Friedel–Crafts addition of the aromatic ring to the gold activated allene. Therefore, selective activation of the alkyne over the allene was considered as a significant challenge in gold catalysis.

## Results and Discussions

**Scheme 3:**
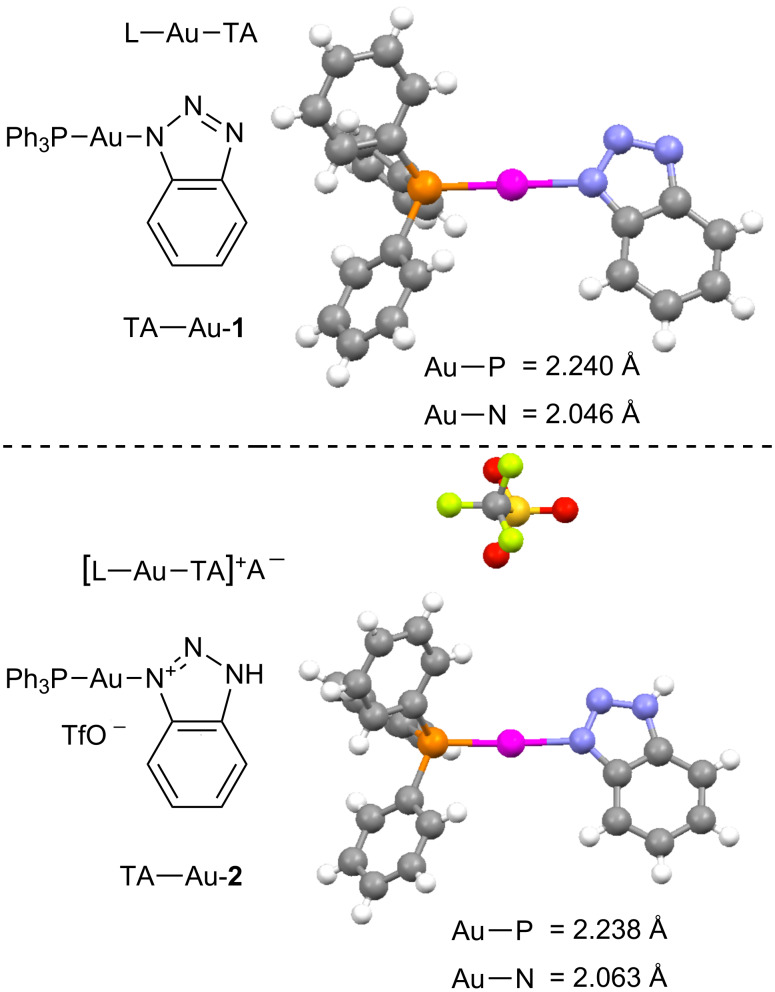
X-ray crystal structures of the two different types of 1,2,3-triazole–Au complexes.

Recently, our group reported the synthesis and characterization of the 1,2,3-triazole [36–40] coordinated gold(I) complexes. As revealed by the X-ray crystal structures (Scheme 3), both neutral and anionic triazoles can coordinate with Au(I) cation, forming stable TA–Au complexes [41].

**Scheme 4:**
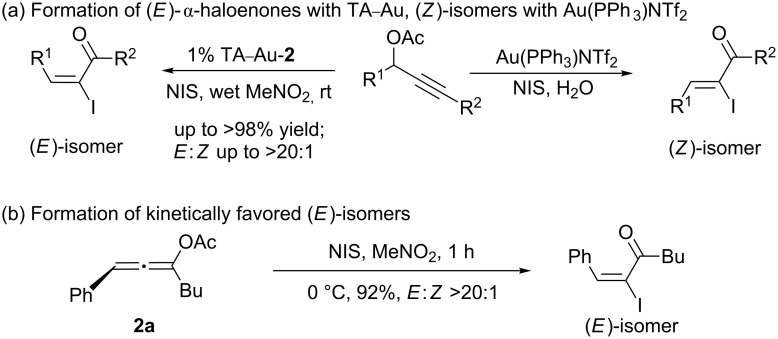
Synthesis of α-iodoenone compounds from propargyl esters.

The preparation of these complexes was very straightforward. Simply treating the NH-triazoles with PPh_3_AuCl in methanol under basic conditions (K_2_CO_3_, 1 equiv) at room temperature gave the neutral TA–Au-**1** in >90% yield. The “cationic” complex TA–Au-**2** was prepared either from the addition of HOTf to TA–Au-**1** or by the reaction between PPh_3_Au–OTf (prepared from PPh_3_–Au–Cl and AgOTf) and benzotriazole. Both complexes were stable and could be further purified by recrystallization to ensure no extra Ag^+^ or acid in the catalysts. The crystal structures revealed nearly identical Au–P bond length for both the anionic and neutral triazole coordinated Au(I) complexes. The longer Au–N bond in TA–Au-**2** implies that the neutral triazole dissociates more easily to release the coordination site for substrate activation. This new class of compounds offers improved thermal stability and substrate stability in the gold(I) promoted hydroamination and Hashmi phenol synthesis [42], which makes them interesting novel catalysts in the field of gold catalysis. One particular new development of the TA–Au catalysis that attracted our attention was the synthesis of α-iodoenone from propargyl esters (Scheme 4a) [43].

**Figure 1:**
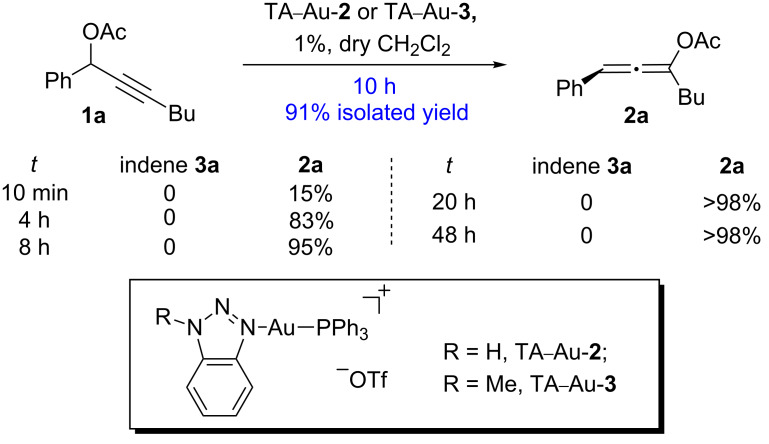
Chemoselective activation of alkyne over allene by the TA–Au catalysts.

As indicated in Scheme 4a, the typical [L–Au]^+^ catalyst promoted the sequential rearrangement and iodination, giving the thermally, dynamically stable (*Z*)-isomer [44–46]. The cationic TA–Au catalyst, on the other hand, produced the kinetically favored (*E*)-isomer. Notably, treating the allene ester **2a** with NIS gave the (*E*)-isomer as the dominant product. These results imply that the allene iodination should favor the formation of the (*E*)-isomer (Scheme 4b). The typical [L–Au]^+^ catalyst not only promoted the propargyl ester 3,3-rearrangement, but also influenced the allene reactivity, probably through gold catalyzed allene activation. The fact that TA–Au gave the dominant (*E*)-isomers strongly suggests that these complexes may be applied as the chemoselective catalyst in alkyne activation over allene. The reactions of propargyl ester **1a** with TA–Au catalysts were then investigated as shown in Figure 1.

As expected, with the cationic catalyst TA–Au-**2** or TA–Au-**3**, the allene ester **2a** was formed in excellent yields (1% loading, 91% yield). It is important to note here, that indene **3a** was not observed even after 48 h reaction time, thus indicating excellent chemoselectivity of the triazole coordinated gold complexes. Various propargyl esters were synthesized to test the reaction substrate scope. The results are summarized in Table 1.

**Table 1:** The reaction substrate scope.^a^

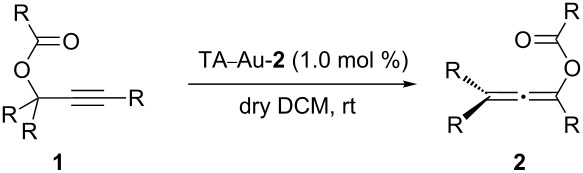					

Entry	Substrate		Product		Yield

1	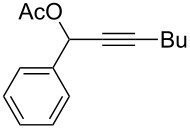	**1a**	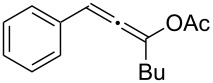	**2a**	91%
2	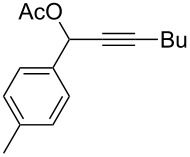	**1b**	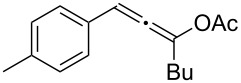	**2b**	90%
3	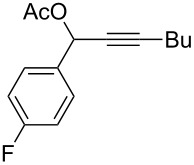	**1c**	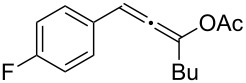	**2c**	87%
4	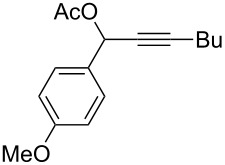	**1d**	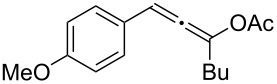	**2d**	89%
5	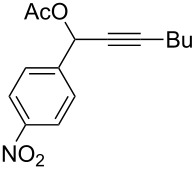	**1e**	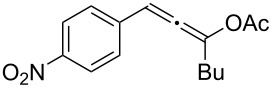	**2e**	89%
6	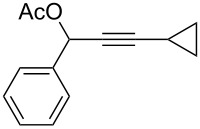	**1f**	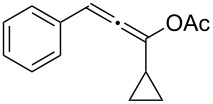	**2f**	85%
Substrates that did not form the desired allenes^b^					
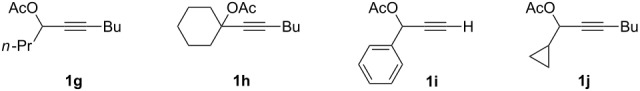					

^a^General reaction conditions: **1** (0.25 mmol) and TA–Au-**2** (1.0 mol %) in dry DCM (2.5 mL), the reactions were monitored by TLC (2–10 h), rt. ^b^TA–Au-**1**, TA–Au-**2** and TA–Au-**3** did not catalyze the reaction under the standard conditions.

As shown in Table 1, the transformation proceeded smoothly with substrates having both an aromatic group on the propargyl side and an aliphatic group on the alkyne side (entries 1–6). The desired allene products were formed in excellent yields, with 1% catalyst loading. The electronic density on the aromatic ring did not have a strong impact on the transformation: Both electron donating and electron-withdrawing groups were suitable for the reaction. Again, no indene by-products were observed in any of the tested cases, even with the electron-enriched *p*-OMe substituted alkyne **1d**. These results highlighted the excellent chemoselective nature of the TA–Au catalyst.

The terminal alkyne **1i** did not give any product when treated with TA–Au catalyst, even after an extended reaction time (24 h). This was probably caused by the preferred 1,2-rearrangement with the formation of a vinyl–Au intermediate. The aliphatic propargyl esters (**1g**, **1h**) also did not give any desired allene products (enones from hydrations were produced after a long reaction time, 24–48 h; the crude NMR of the reaction mixtures did not show any allene products). This may be caused by the high reactivity of the corresponding aliphatic allenes under the reaction conditions (activated by TA–Au) and the overall better stability of the propargyl ester compared to the aliphatic substituted allenes (equilibrium favored the starting material). The reaction of cyclopropyl substituted propargyl ester **1j** with the TA–Au catalyst gave a complex reaction mixture, which suggests possible ring opening and sequential cyclization as reported previously [47]. Overall, this study suggests that the propargyl ester rearrangement to form allene is highly substrate dependent. This could either be due to the similar reactivity of the alkyne and the allene (giving an equilibrium state favoring the alkyne over the allene) or it be could be due to a preferred alternative migration path (2,3-migration versus 3,3-migration). In any case, the TA–Au catalyst clearly displayed the interesting chemoselectivity, if the reaction could occur. To study the feasibility of this migration, we then investigated migrating groups other than esters. The results are summarized in Table 2.

**Table 2:** Different migrating groups.^a^

Entry	Substrate		Product		Yield

1	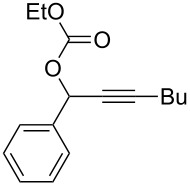	**4a**	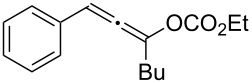	**5a**	92%
2	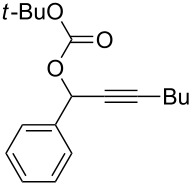	**4b**	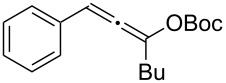	**5b**	91%
3	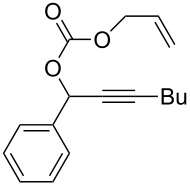	**4c**	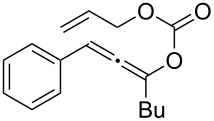	**5c**	88%
4	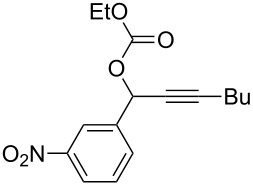	**4d**	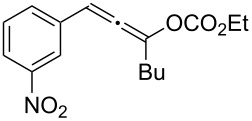	**5d**	92%
5	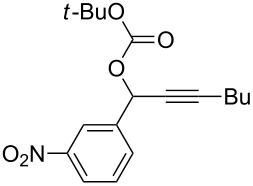	**4e**	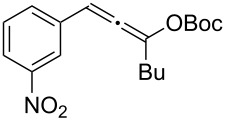	**5e**	89%
6	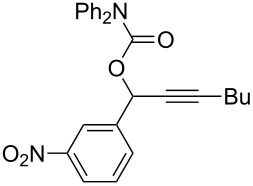	**4f**	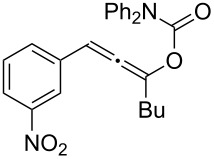	**5f**	85%

^a^General reaction condition: **4** (0.25 mmol) and TA–Au-**2** (1.0 mol %) in dry DCM (2.5 mL), the reactions were monitored by TLC (2–10 h), rt.

As indicated in Table 2, carbonates (entries 1–5) and carbamate (entry 6) were also suitable for this transformation. Compared to the allene-acetates, the allene-carbonates and allene-carbamates were more stable in water. Notably, although the alkene was considered as a readily reactive functional group in gold catalysis, the substrate **4c** was suitable for this transformation, giving the desired allene-ene **5c** in excellent yield.

## Conclusion

In this letter, we reported the application of triazole-coordinated gold(I) complexes as the effective catalysts for the promotion of the propargyl ester, carbonate and carbamate 3,3-rearrangement for the synthesis of the corresponding substituted allene derivatives. The chemoselective nature of the TA–Au catalysts was clearly demonstrated, which makes them an interesting class of new catalysts for promoting organic transformations. The application of the allene-carbonates and allene-carbamates as building blocks for development of new synthetic methodologies is currently underway in our group.

## Supporting Information

File 1 General methods, characterization data and NMR spectra of synthesized compounds.
