# Supplementary material for: Triazole–Au(I) complex as chemoselective catalyst in promoting propargyl ester rearrangements
Source: Beilstein J Org Chem. 2011 Jul 25;7:1014–20. doi: 10.3762/bjoc.7.115 (PMC3167893; doi:10.3762/bjoc.7.115)
Supplement: File 1 — General methods, characterization data and NMR spectra of synthesized compounds. [file Beilstein_J_Org_Chem-07-1014-s001.pdf]

Supporting Information

for

Triazole–Au(I) complex as chemoselective catalyst in promoting propargyl ester rearrangements

Dawei Wang, Yanwei Zhang, Rong Cai and Xiaodong Shi*

Address: Department of Chemistry, West Virginia University, Morgantown, WV 26506, USA

Email: Xiaodong Shi* - Xiaodong.Shi@mail.wvu.edu

* Corresponding author

General methods, characterization data and NMR spectra of synthesized compounds.

1. General methods and materials **S2**

2. Compounds characterization **S3-S6**

3. References **S6**

4. NMR spectra **S7-S30**

1. General methods and materials:

All of the reactions dealing with air and/or moisture-sensitive react were carried out under an atmosphere of nitrogen using oven/flame-dried glassware and standard syringe/septa techniques. Unless otherwise noted, all commercial reagents and solvents were obtained from a commercial provider and used without further purification. ^{1}H NMR and ^{13}C NMR spectra were recorded on Varian 600 MHz spectrometers. Chemical shifts were reported relative to internal tetramethylsilane (δ 0.00 ppm) or $CDCl_3$ (δ 7.26 ppm) for ^{1}H NMR and $CDCl_3$ (δ 77.0 ppm) for ^{13}C NMR. Flash column chromatography was performed on 230–430 mesh silica gel. Analytical thin layer chromatography was performed with precoated glass baked plates (250µ) and visualized by fluorescence and by charring after treatment with potassium permanganate stain. HRMS were recorded on LTQ-FTUHRA spectrometer.

Substrates **1** and **5** were synthesized according to the literature [1-3].

Representative procedure for the preparation of allene 2a

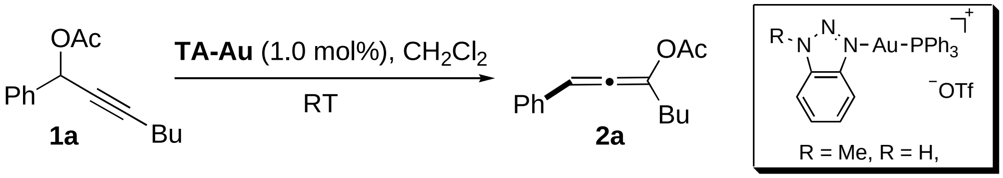

To a solution of **1a** (58 mg, 0.25 mmol) in dry CH_2Cl_2 (2.5 mL, 0.1 M), was added Au(I) catalyst (1.9 mg, 0.0025 mol, 1.0 mol %) at RT. The reaction mixture was stirred at RT and monitored by TLC. After the reaction was completed (2–10 h), the solvent was removed under reduced pressure and the residue was purified by flash chromatography on silica gel (ethyl acetate/hexane = 1 : 20, v/v) to give **2a** (91% yield) as colorless oil.

2. Compounds characterization

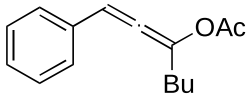

2a

1-Phenylhepta-1,2-dien-3-yl acetate (2a): (Known compound, see [4].) 91% yield, ^1H NMR (600 MHz, $CDCl_3$): δ 7.43-7.44 (m, 2H), 7.32-7.35 (m, 2H), 7.25-7.27 (m, 1H), 6.59 (t, J = 3.0 Hz, 1H), 2.33-2.37 (m, 2H), 2.15 (s, 3H), 1.46-1.51 (m, 2H), 1.38-1.43 (m, 2H), 0.90 (t, J = 7.2 Hz, 3H); ^{13}C NMR (150 MHz, $CDCl_3$): δ 196.7, 168.6, 133.9, 129.2, 128.6, 127.9, 127.0, 104.5, 31.5, 28.3, 22.1, 21.0, 13.8.

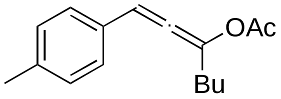

2b

1-*p*-Tolylhepta-1,2-dien-3-yl acetate (**2b**): (Known compound, see [4].) 90% yield, ^1H NMR (600 MHz, $CDCl_3$): δ 7.32 (d, J = 8.4 Hz, 2H), 7.13 (d, J = 7.8 Hz, 2H), 6.56 (t, J = 3.0 Hz, 1H), 2.31-2.35 (m, 2H), 2.33 (s, 3H), 2.13 (s, 3H), 1.45-1.48 (m, 2H), 1.34-1.40 (m, 2H), 0.89 (t, J = 7.2 Hz, 3H); ^{13}C NMR (150 MHz, $CDCl_3$): δ 196.1, 168.6, 137.9, 131.0, 129.3, 127.7, 126.7, 104.4, 31.5, 28.3, 22.1, 21.2, 21.0, 13.8.

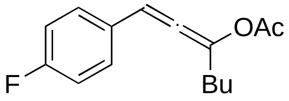

2c

1-(4-Fluorophenyl)hepta-1,2-dien-3-yl acetate (**2c**): (Known compound, see [4].) 87% yield, ^1H NMR (600 MHz, $CDCl_3$): δ 7.39-7.41 (m, 2H), 7.00-7.03 (m, 2H), 6.55 (t, J = 3.0 Hz, 1H), 2.31-2.35 (m, 2H), 2.15 (s, 3H), 1.44-1.49 (m, 2H), 1.36-1.41 (m, 2H), 0.90 (t, J = 7.2 Hz, 3H). ^{13}C NMR (150 MHz, $CDCl_3$): δ 196.4, 168.6, 161.8 (d, J = 246.5 Hz), 130.0 (d, J = 3.2 Hz), 129.4 (d, J = 8.3 Hz), 126.9, 115.5 (d, J = 21.9 Hz), 103.4 (d, J = 2.7 Hz), 31.5, 28.3, 22.1, 21.0, 13.8.

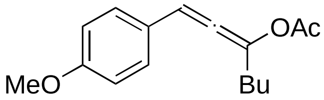

2d

1-(4-Methoxyphenyl)hepta-1,2-dien-3-yl acetate (**2d**): (Known compound, see [4].) 89% yield, ^1H NMR (600 MHz, $CDCl_3$): δ 7.36 (d, J = 8.4 Hz, 2H), 6.87 (d, J = 9.0 Hz, 2H), 6.54 (t, J = 3.0 Hz, 1H), 3.81 (s, 3H), 2.28-2.34 (m, 2H), 2.14 (s, 3H), 1.44-1.47 (m, 2H), 1.35-1.39 (m, 2H), 0.90 (t, J = 7.2 Hz, 3H). ^{13}C NMR (150 MHz, $CDCl_3$): δ 195.3, 168.8, 159.6, 129.0, 126.6, 126.4, 114.2, 104.0, 55.3, 31.6, 28.4, 22.1, 21.1, 13.8.

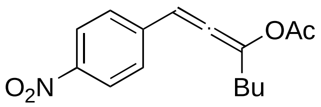

2e

1-(4-Nitrophenyl)hepta-1,2-dien-3-yl acetate (**2e**): 89% yield, ^{1}H NMR (600 MHz, $CDCl_3$): δ 8.18-8.20 (m, 2H), 7.56-7.58 (m, 2H), 6.63 (t, *J* = 3.0 Hz, 1H), 2.34-2.37 (m, 2H), 2.17 (s, 3H), 1.45-1.55 (m, 2H), 1.37-1.41 (m, 2H), 0.91 (t, *J* = 7.2 Hz, 3H). ^{13}C NMR (150 MHz, $CDCl_3$): δ 200.2, 168.2, 147.2, 140.9, 128.3, 127.5, 124.0, 102.6, 31.4, 28.2, 22.1, 22.0, 13.8. HRMS Calculated for $[C_{15}H_{17}NO_4+Na]^+$: 298.1050, Found: 298.1050.

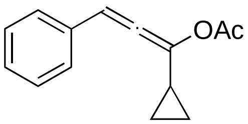

2f

1-Cyclopropyl-3-phenylpropa-1,2-dienyl acetate (**2f**): 85% yield, ^{1}H NMR (600 MHz, $CDCl_3$): δ 7.39-7.40 (m, 2H), 7.31-7.33 (m, 2H), 7.23-7.24 (m, 1H), 6.59 (d, *J* = 2.4 Hz, 1H), 2.16 (s, 3H), 1.55-1.59 (m, 1H), 0.77-0.80 (m, 2H), 0.59-0.65(m, 2H). ^{13}C NMR (150 MHz, $CDCl_3$): δ 196.4, 168.6, 133.7, 128.7, 128.1, 127.8, 105.1, 93.0, 20.9, 11.9, 6.4, 5.9. HRMS Calculated for $[C_{14}H_{14}O_2+H]^+$: 215.1066, Found: 215.1059.

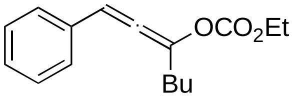

5a

Ethyl 1-phenylhepta-1,2-dien-3-yl carbonate (5a): 92% yield, ^{1}H NMR (600 MHz, $CDCl_3$): δ 7.42-7.43 (m, 2H), 7.32-7.35 (m, 2H), 7.24-7.27 (m, 1H), 6.65 (t, *J* = 3.0 Hz, 1H), 4.24 (q, *J* = 7.2 Hz, 2H), 2.37-2.41 (m, 2H), 1.47-1.51 (m, 2H), 1.37-1.41 (m, 2H), 1.32 (t, *J* = 7.2 Hz, 3H), 0.89 (t, *J* = 7.2 Hz, 3H); ^{13}C NMR (150 MHz, $CDCl_3$): δ 196.4, 153.1, 133.7, 128.7, 128.3, 128.1, 127.9, 105.7, 64.5, 31.3, 28.2, 22.1, 14.2, 13.8. HRMS Calculated for $[C_{16}H_{20}O_3+Na]^+$: 283.1305, Found: 283.1305.

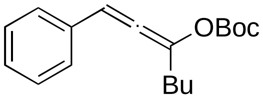

5b

***t*-Butyl 1-phenylhepta-1,2-dien-3-yl carbonate** (**5b**): 91% yield, ^{1}H NMR (600 MHz, $CDCl_3$): δ 7.41-7.42 (m, 2H), 7.31-7.34 (m, 2H), 7.23-7.26 (m, 1H), 6.63 (t, *J* = 3.0 Hz, 1H), 2.35-2.39 (m, 2H), 1.50 (s, 9H), 1.46-1.48 (m, 2H), 1.37-1.41 (m, 2H), 0.89 (t, *J* = 7.5 Hz, 3H). ^{13}C NMR (150 MHz, $CDCl_3$): δ 196.7, 151.2, 134.0, 128.6, 128.1, 128.0, 127.8, 105.2, 82.9, 31.4, 28.3, 27.9, 22.1, 13.8. HRMS Calculated for $[C_{18}H_{24}O_3+Na]^+$: 311.1618, Found: 311.1604.

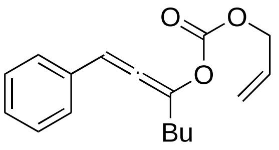

5c

Allyl 1-phenylhepta-1,2-dien-3-yl carbonate (5c): 88% yield, ^{1}H NMR (600 MHz, $CDCl_3$): δ 7.41 (d, *J* = 7.8 Hz, 2H), 7.33 (t, *J* = 7.5 Hz, 2H), 7.24-7.27 (m, 1H), 6.65 (s, 1H), 5.91-5.97 (m, 1H), 5.35 (d, *J* = 17.4 Hz, 1H), 5.26 (d, *J* = 10.2 Hz, 1H), 4.65 (d, *J* = 6.0 Hz, 2H), 2.30-2.34 (m, 2H), 1.47-1.53 (m, 2H), 1.36-1.43 (m, 2H), 0.89 (t, *J* = 6.9 Hz, 3H). ^{13}C NMR (150 MHz, $CDCl_3$): δ 196.3, 152.9, 133.7, 131.3, 128.7, 128.4, 128.1, 127.9, 119.2, 105.8, 68.9, 31.3, 28.2, 22.1, 13.8. HRMS Calculated for $[C_{17}H_{20}O_3+Na]^+$: 295.1305, Found: 295.1306.

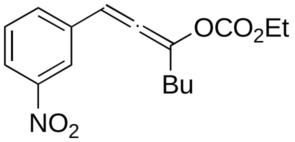

5d

Ethyl 1-(3-nitrophenyl)hepta-1,2-dien-3-yl carbonate (**5d**): 92% yield, ^{1}H NMR (600 MHz, $CDCl_3$): δ 8.25-8.26 (m, 1H), 8.09-8.12 (m, 1H), 7.77-7.78 (m, 1H), 7.51 (t, *J* = 8.1 Hz, 1H), 6.70 (t, *J* = 3.0 Hz, 1H), 4.24 (q, *J* = 7.4 Hz, 1H), 2.39-2.43 (m, 2H), 1.48-1.56 (m, 2H), 1.39-1.44 (m, 2H), 1.33 (t, *J* = 8.1 Hz, 3H), 0.91 (t, *J* = 7.2 Hz, 3H). ^{13}C NMR (150 MHz, $CDCl_3$): δ 198.3, 152.7, 148.7, 135.9, 133.4, 129.5, 129.2, 122.7, 122.5, 103.6, 64.8, 31.3, 28.1, 22.1, 14.2, 13.7. HRMS Calculated for $[C_{16}H_{19}NO_5+Na]^+$: 328.1155, Found: 328.1142.

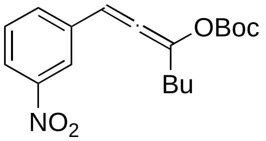

5e

***t*-Butyl 1-(3-nitrophenyl)hepta-1,2-dien-3-yl carbonate** (**5e**): 89% yield, ^{1}H NMR (600 MHz, $CDCl_3$): δ 8.23-8.24 (m, 1H), 8.09-8.11 (m, 1H), 7.77 (dt, *J* = 7.8 Hz, 1.2 Hz, 1H), 7.50 (t, *J* = 8.1 Hz, 1H), 6.68 (t, *J* = 3.0 Hz, 1H), 2.37-2.41 (m, 2H), 1.51 (s, 9H), 1.47-1.50 (m, 2H), 1.38-1.42 (m, 2H), 0.90 (t, *J* = 7.2 Hz, 3H). ^{13}C NMR (150 MHz, $CDCl_3$): δ 198.6, 150.8, 148.7, 136.2, 133.4, 129.5, 128.9, 122.5, 122.4, 103.2, 83.4, 31.4, 28.2, 27.7, 22.1, 13.8. HRMS Calculated for $[C_{18}H_{23}NO_5+Na]^+$: 356.1468, Found: 356.1454.

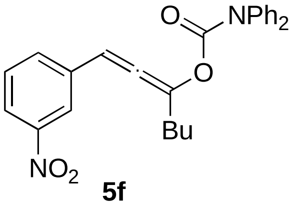

5f

1-(3-Nitrophenyl)hepta-1,2-dien-3-yl diphenylcarbamate (**5f**): 85% yield, ^{1}H NMR (600 MHz, $CDCl_3$): δ 8.22 (t, *J* = 2.1 Hz, 1H), 8.07 (dq, *J* = 8.4 Hz, 1.0 Hz, 1H), 7.77 (dt, *J* = 7.8 Hz, 1.2 Hz, 1H), 7.48 (t, *J* = 7.8 Hz, 1H), 7.34 (t, *J* = 7.8 Hz, 4H), 7.21-7.28 (m, 6H), 6.65 (t, *J* = 3.0 Hz, 1H), 2.30-2.34 (m, 2H), 1.31-1.41 (m, 4H), 0.85 (t, *J* = 7.2 Hz, 3H). ^{13}C NMR (150 MHz, $CDCl_3$): δ 198.7, 152.0, 148.6, 142.2, 136.4, 133.5, 129.5, 128.9, 128.3, 126.8, 126.4, 122.4, 122.3, 102.8, 31.6, 28.1, 21.9, 13.7. HRMS Calculated for $[C_{26}H_{24}N_2O_4+Na]^+$: 451.1628, Found: 451.1610.

3. References

1. Yu, M.; Zhang, G.; Zhang, L. *Org. Lett*. **2007**, *9*, 2147–2150.
2. Marion, N.; Carlqvist, P.; Gealageas, R.; Fremont, P.; Maseras, F.; Nolan, S. P. *Chem. Eur. J*. **2007**, *13*, 6437–6451.
3. Nonoshita, K.; Banno, H.; Maruoka, K.; Yamamoto, H. *J. Am. Chem. Soc.* **1990**, *112*, 316–322.
4. Nun, P.; Gaillard S.; Slawin, A. M. Z.; Nolan, S. P. *Chem. Commun.*, **2010**, *46*, 9113–9115

4. NMR spectra

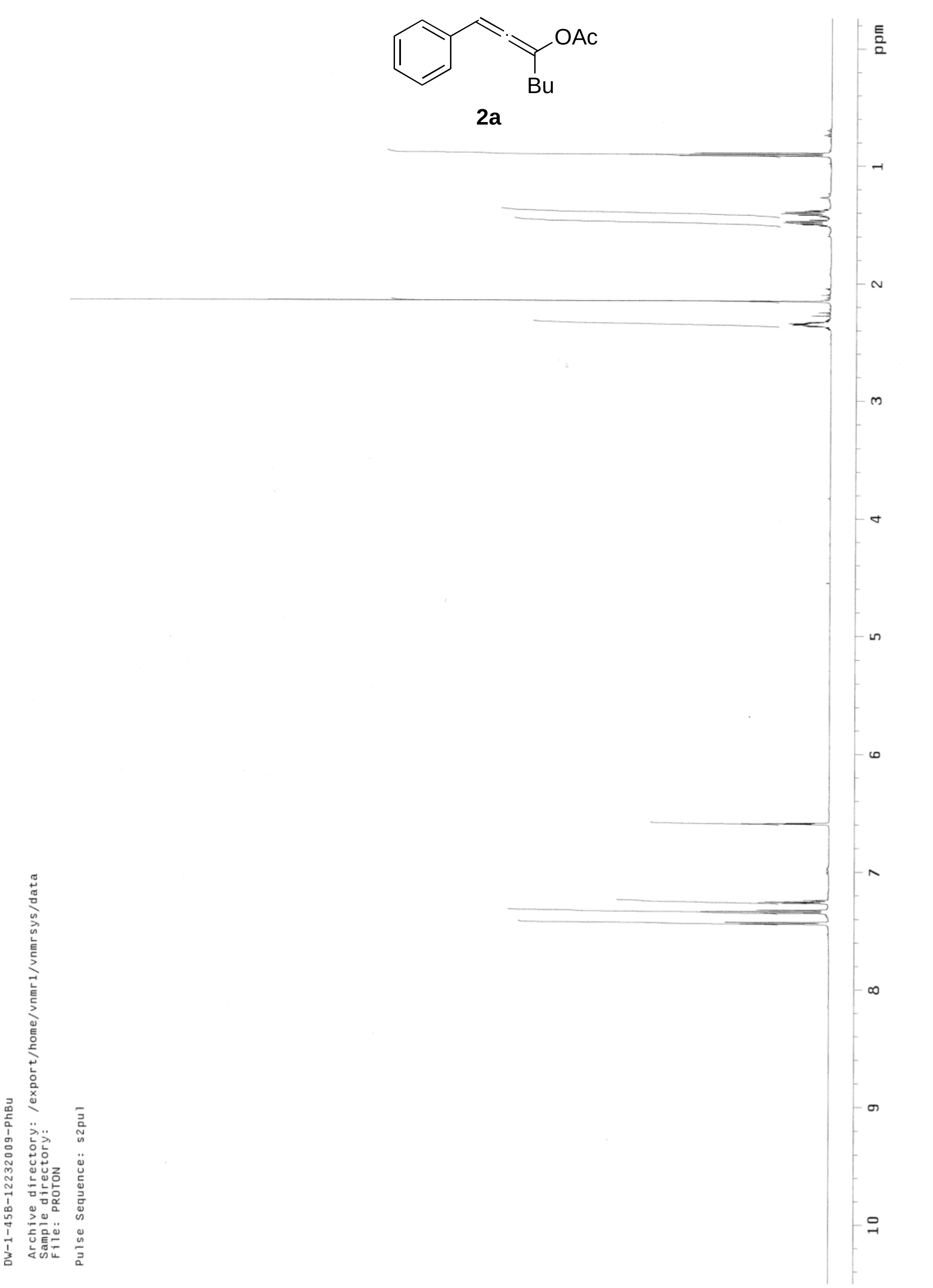

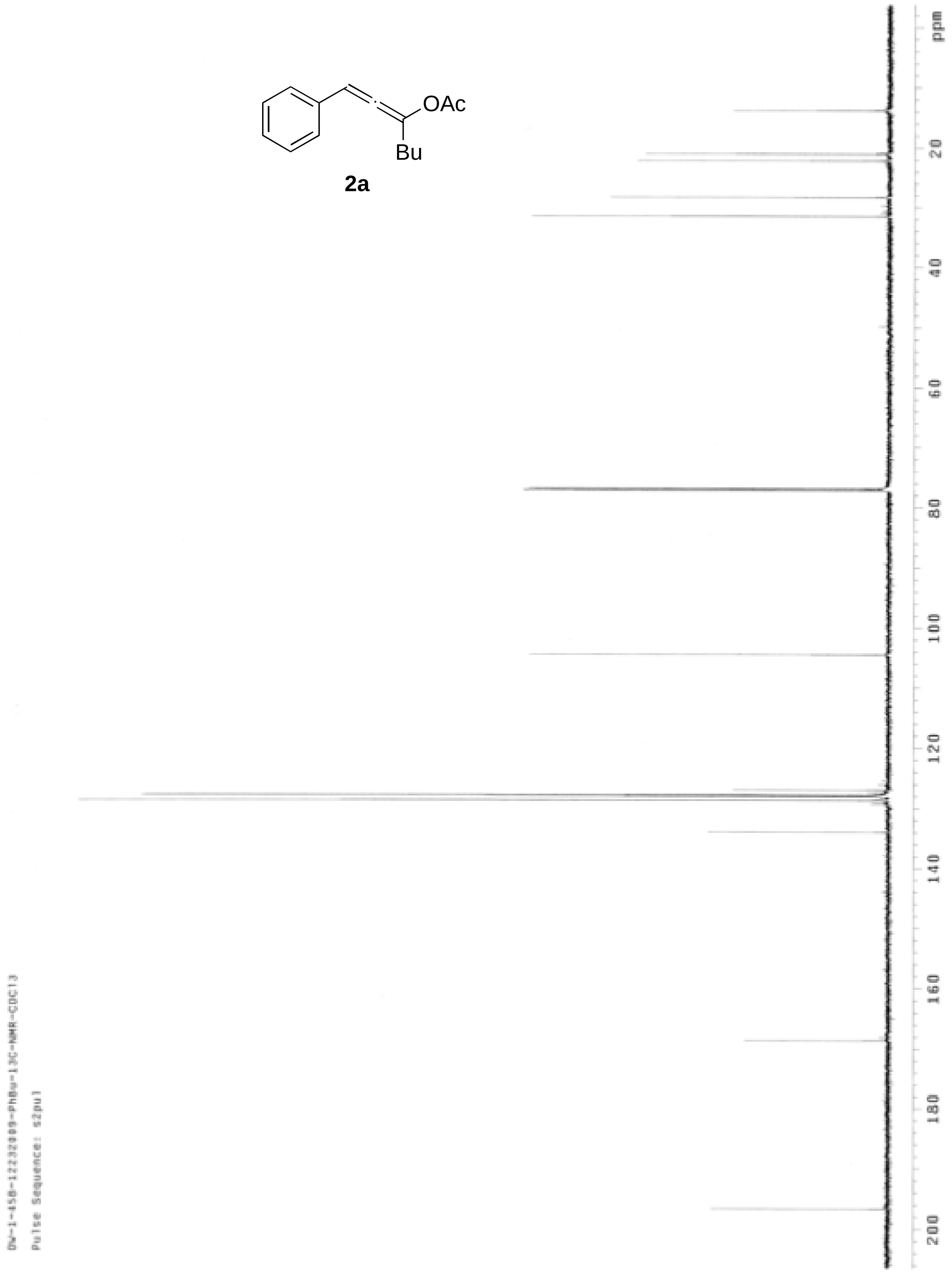
OAc
Bu
2a
Pulse Sequence: s2pul
ppm
20
40
60
80
100
120
140
160
180
200

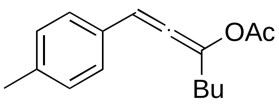

2b, 90%

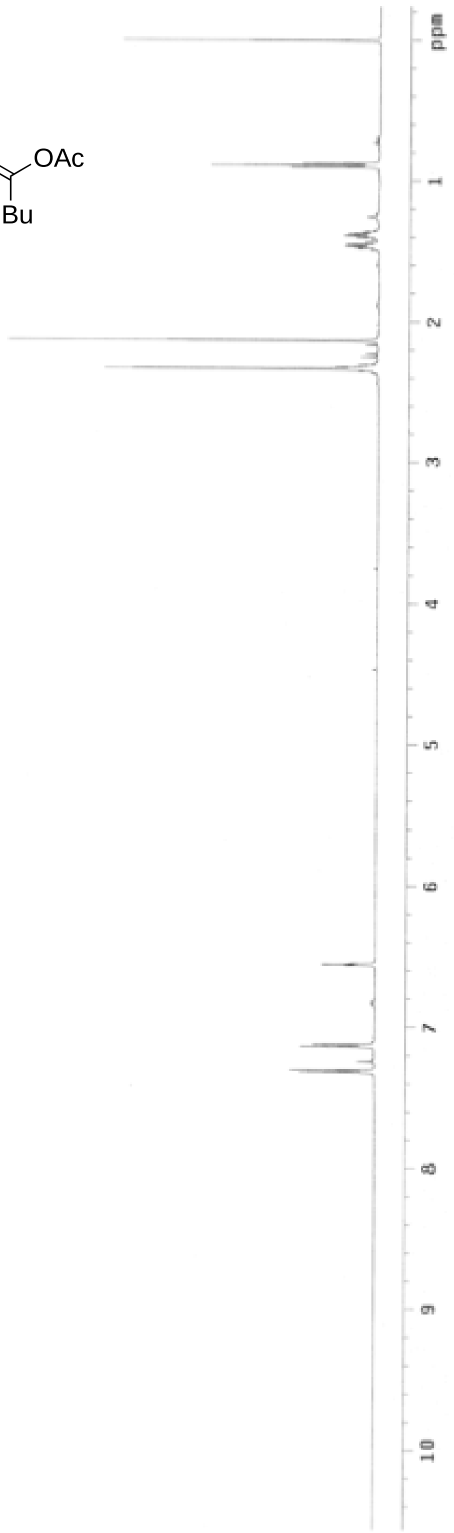

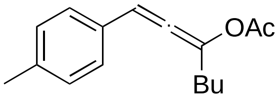

2b, 90%

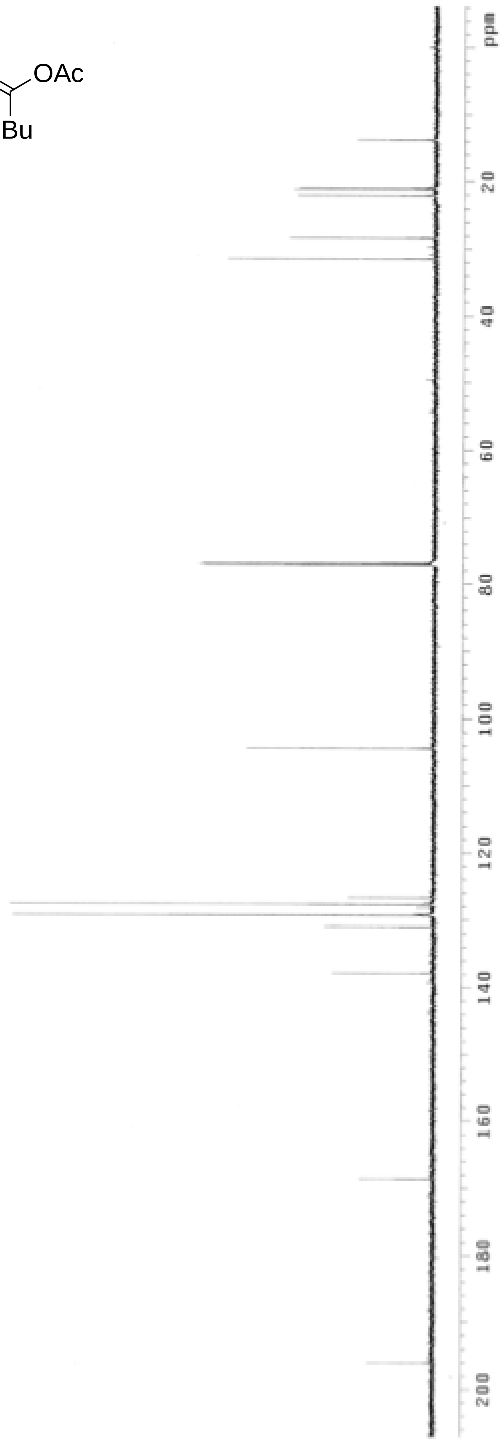

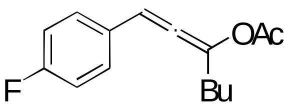

2c

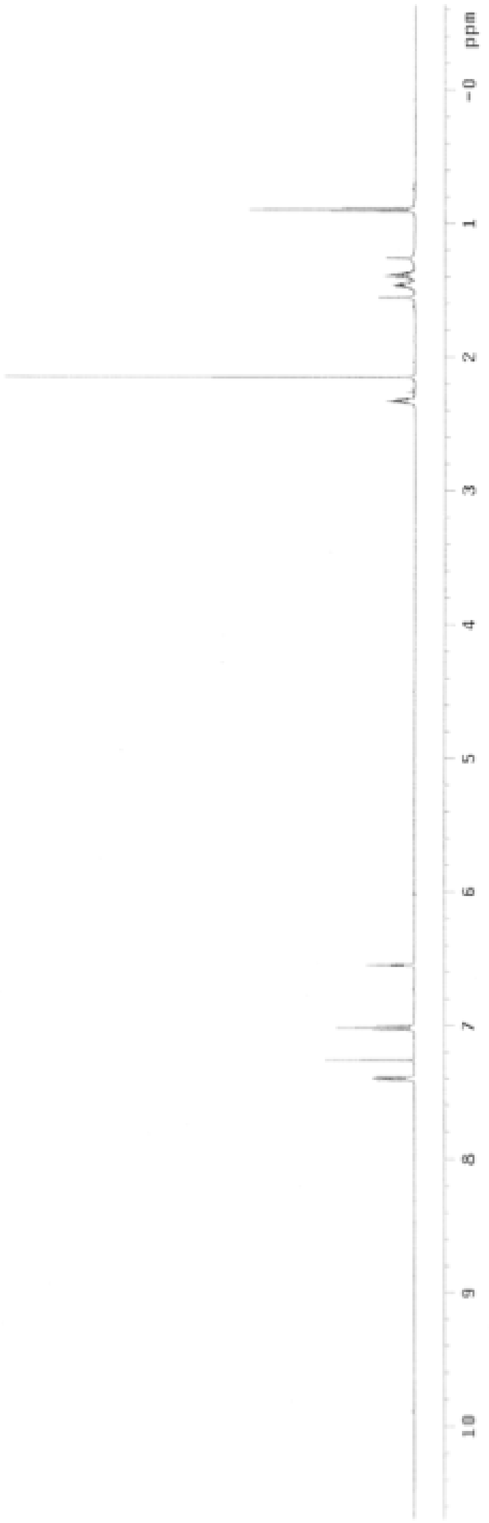

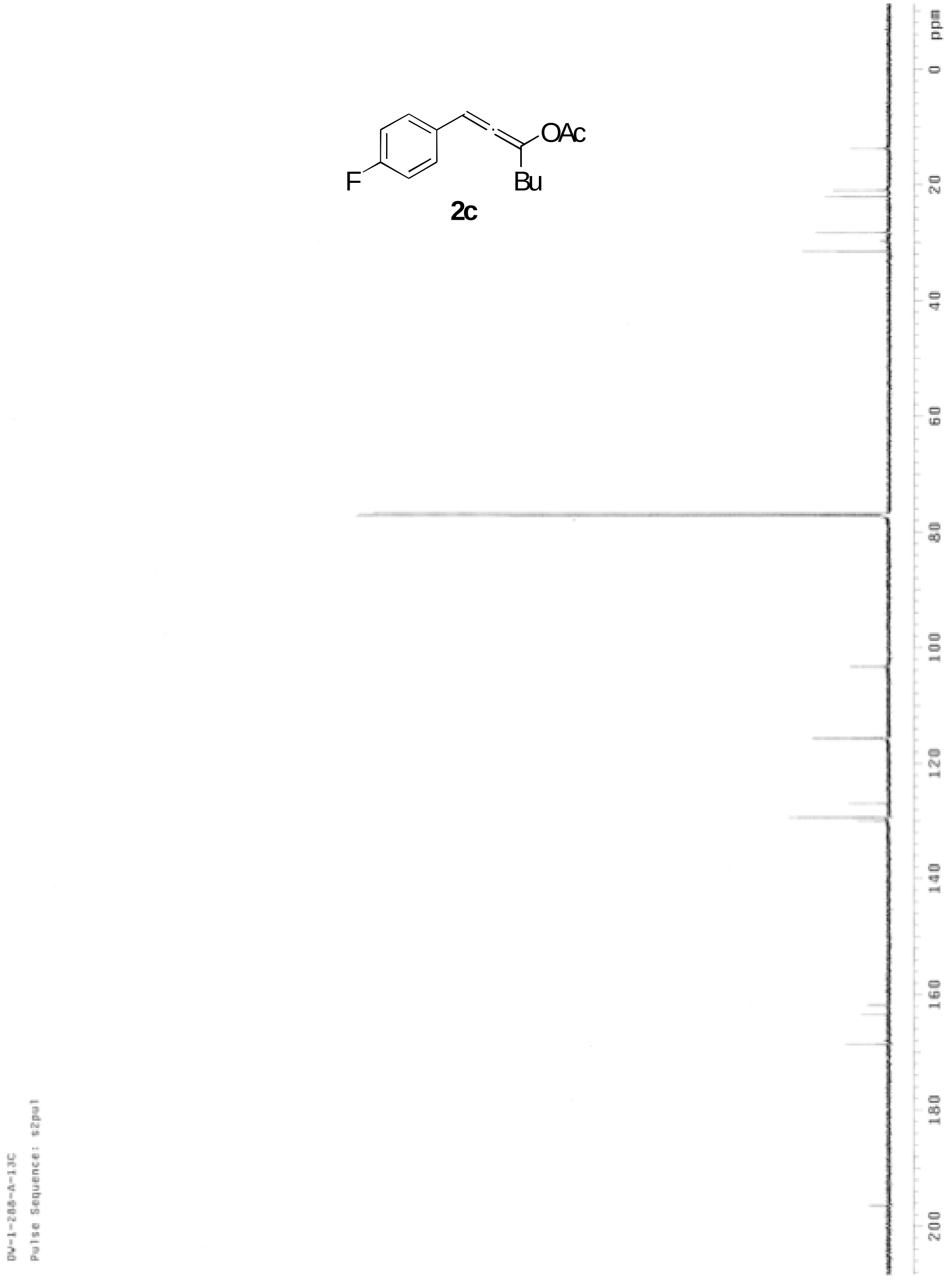
OAc
F
Bu
2c
DV-1-288-A-13C
Pulse Sequence: s2pul
ppm
0
20
40
60
80
100
120
140
160
180
200

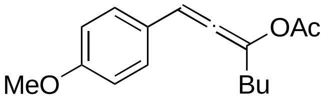

2d, 89%

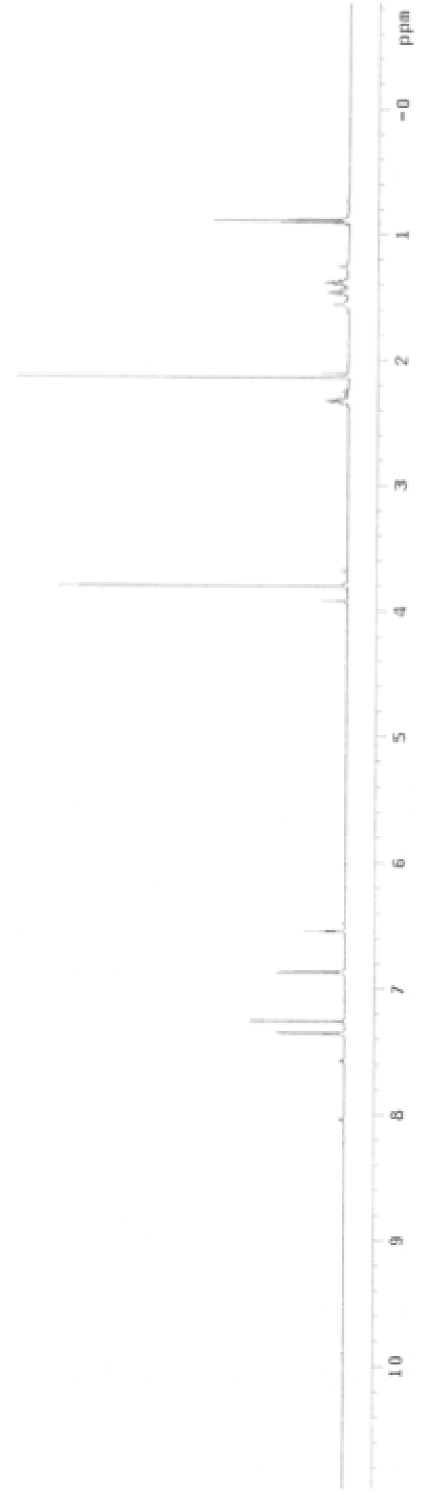

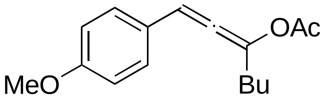

2d, 89%

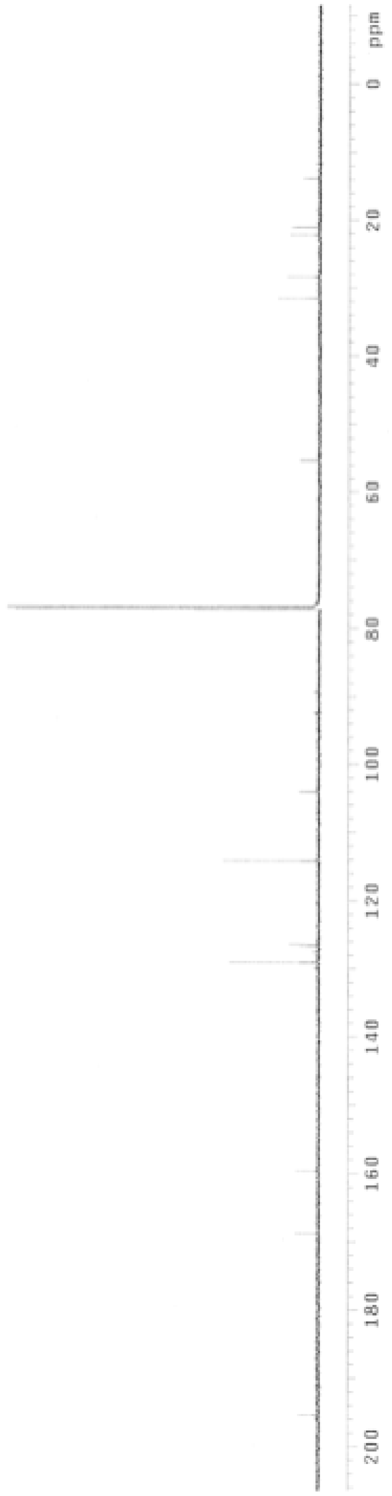

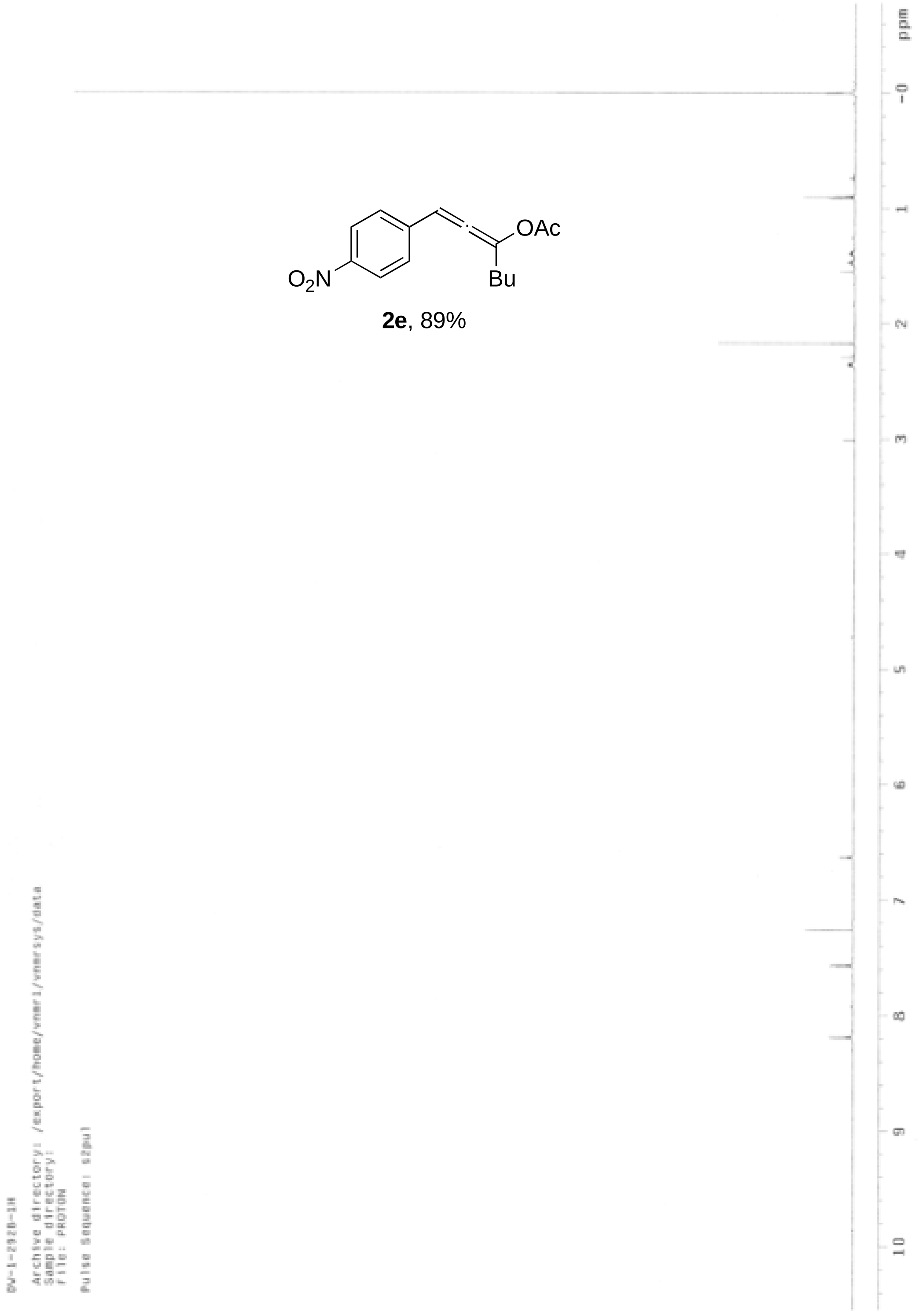

2e, 89%

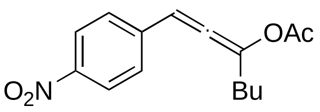

2e, 89%

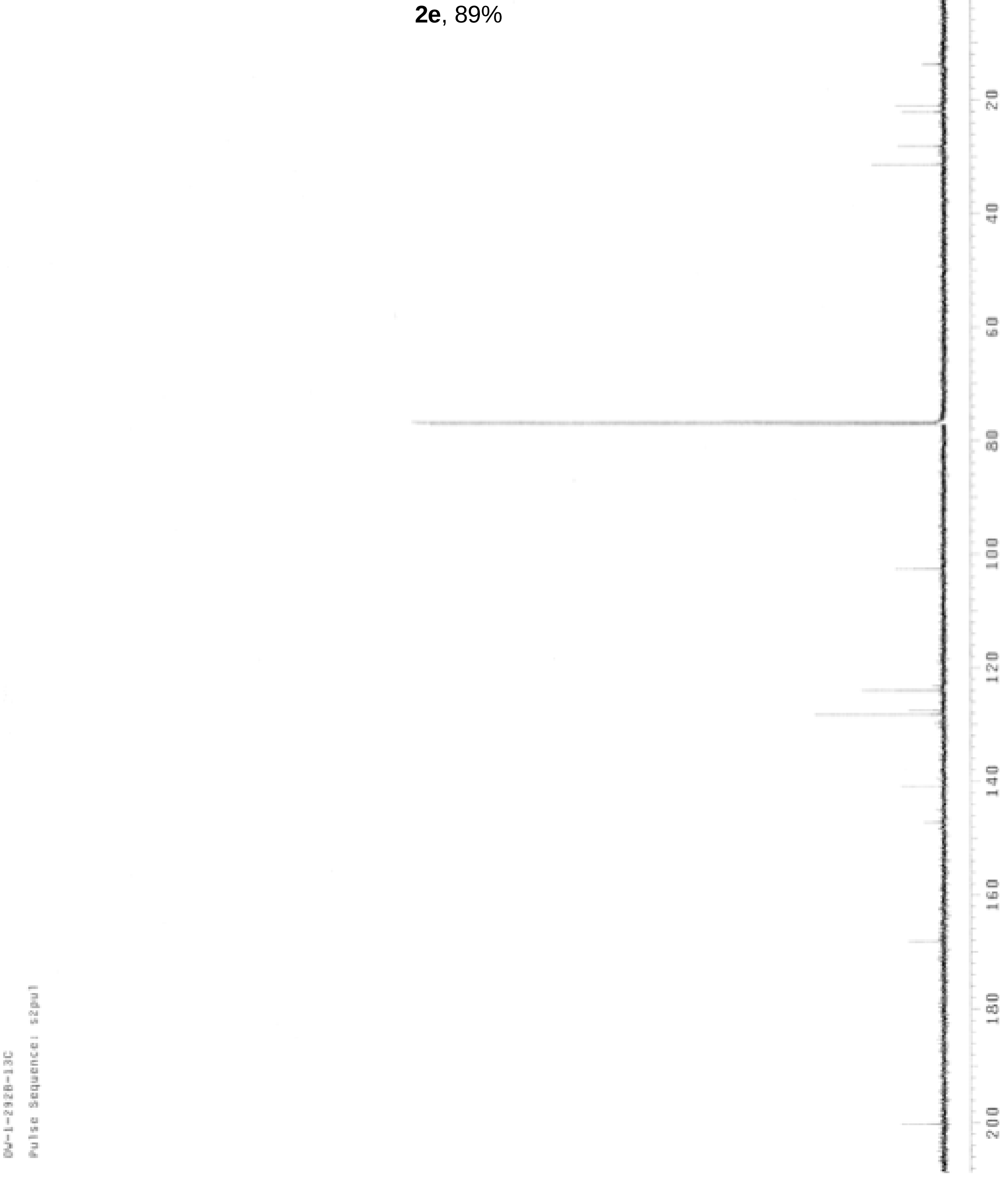

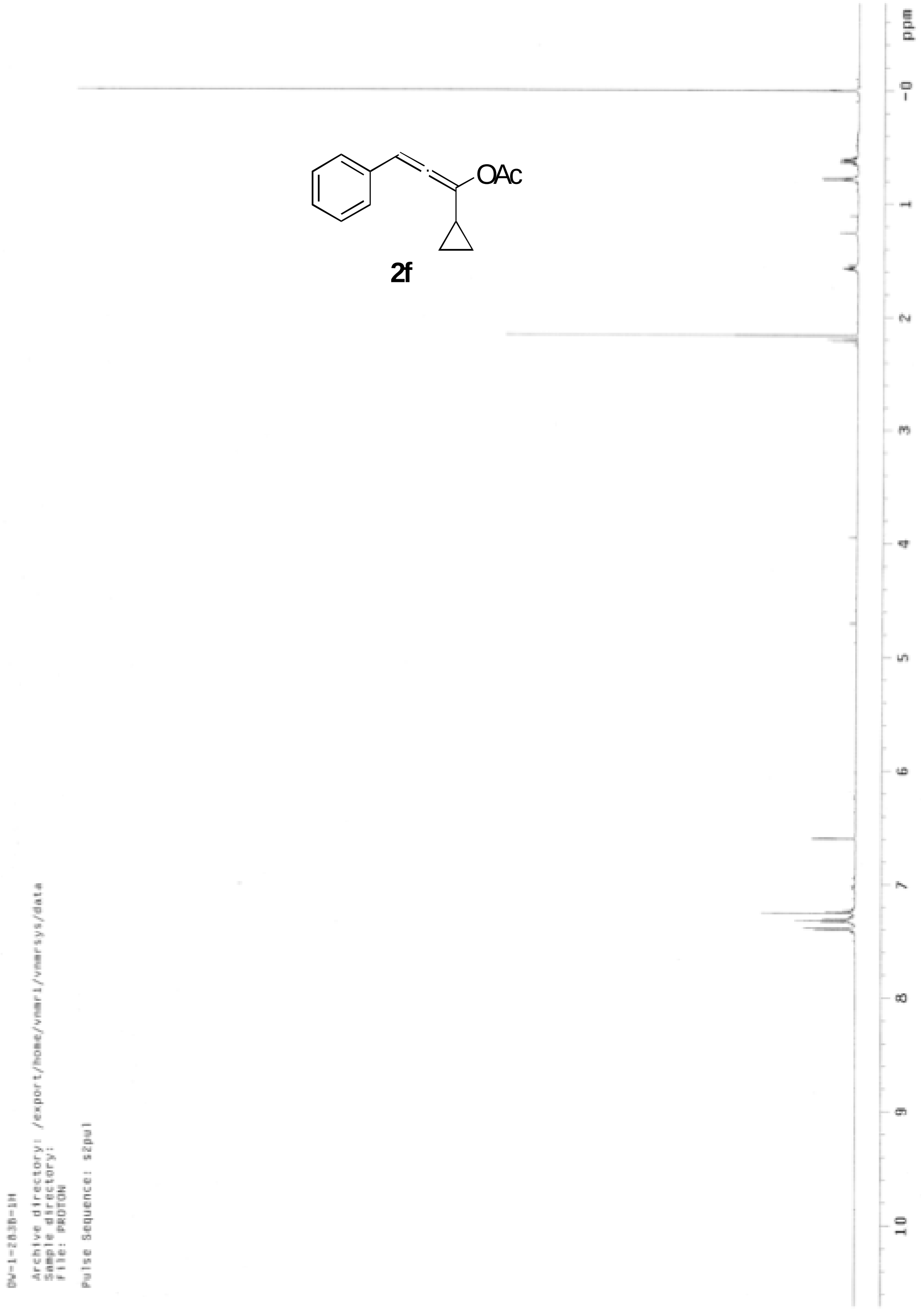
OAc
2f
ppm

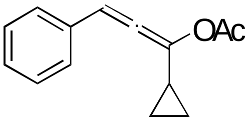
OAc

2f

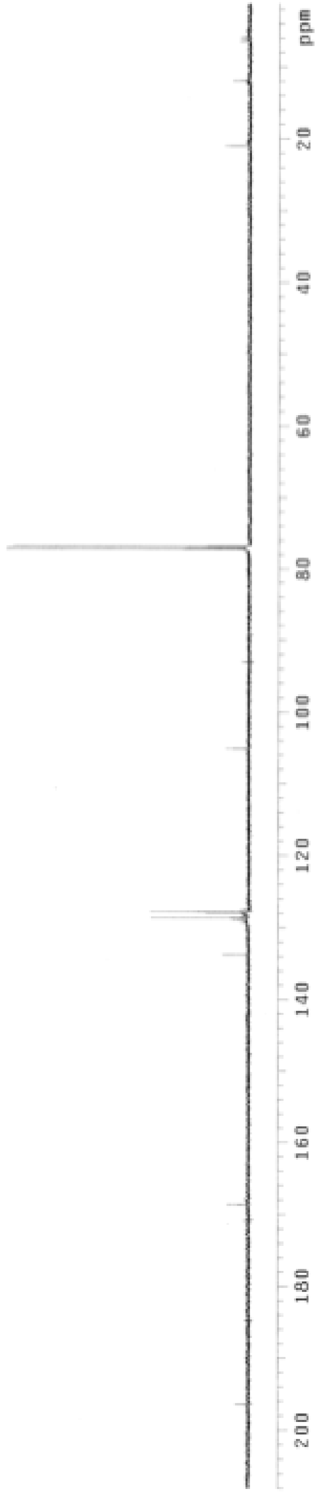
DW-1-283B-13C
Pulse Sequence: s2pul
ppm
20
40
60
80
100
120
140
160
180
200

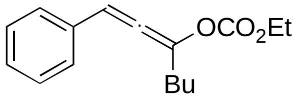

5a, 92%

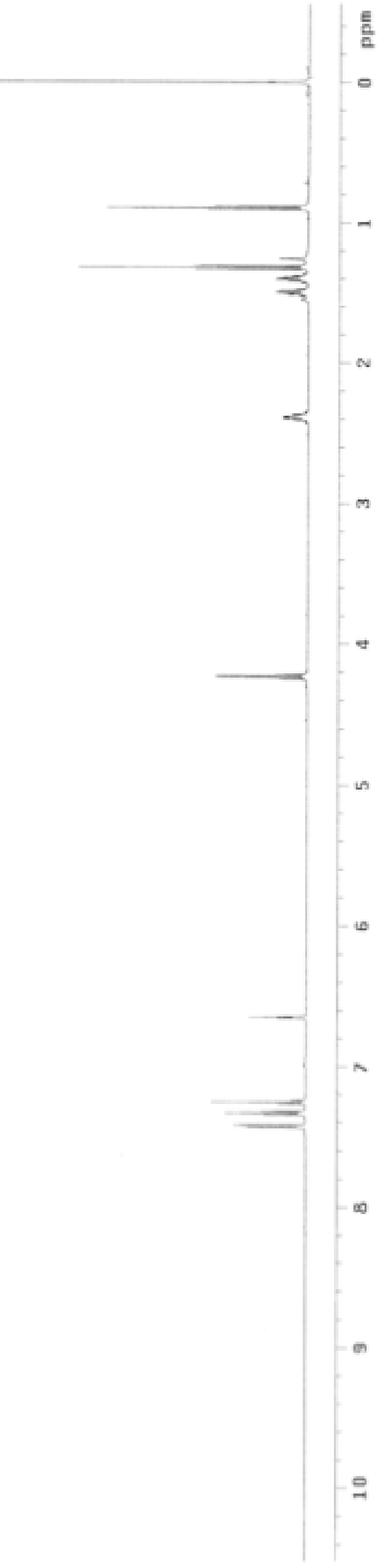

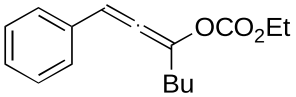

5a, 92%

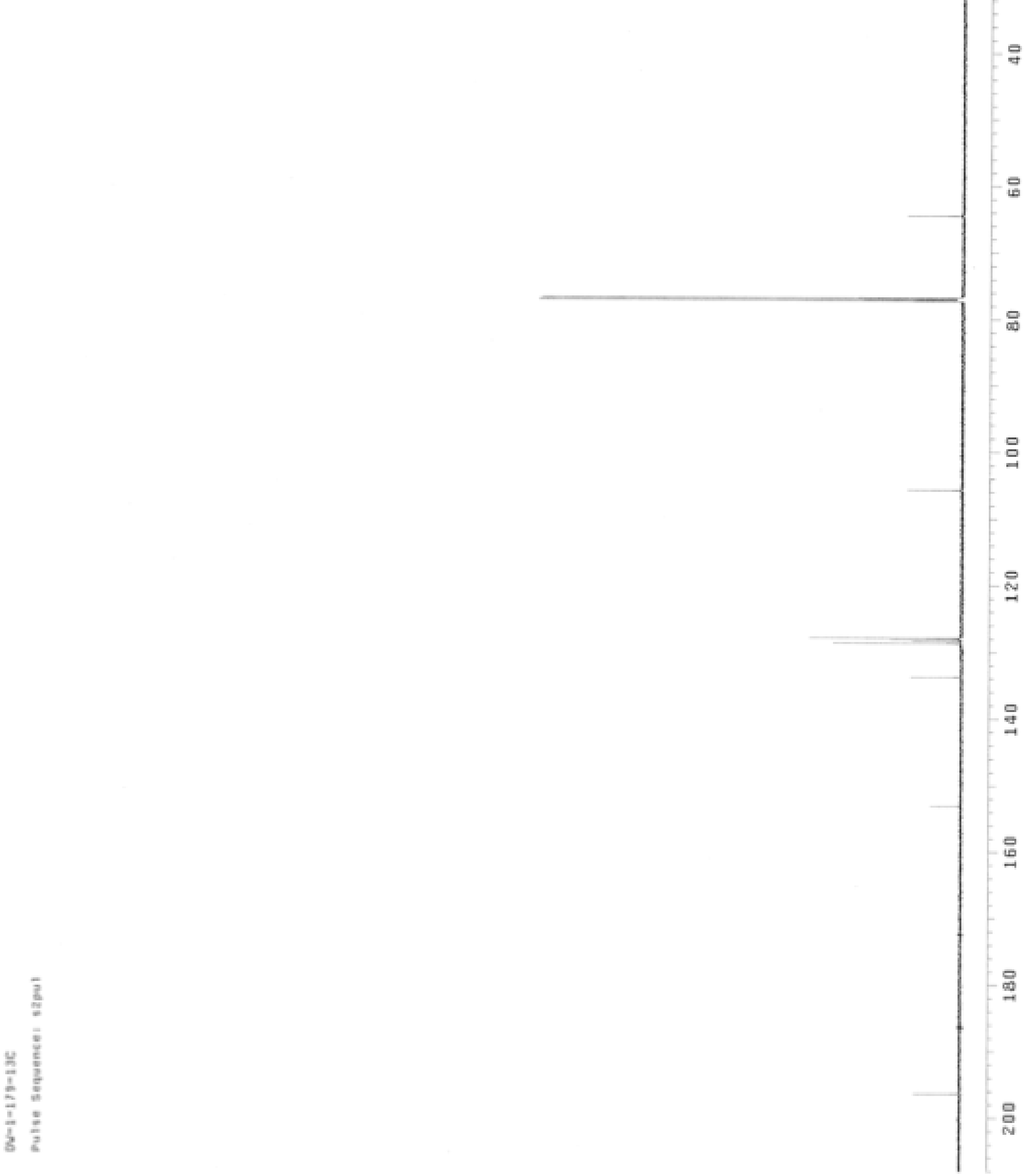

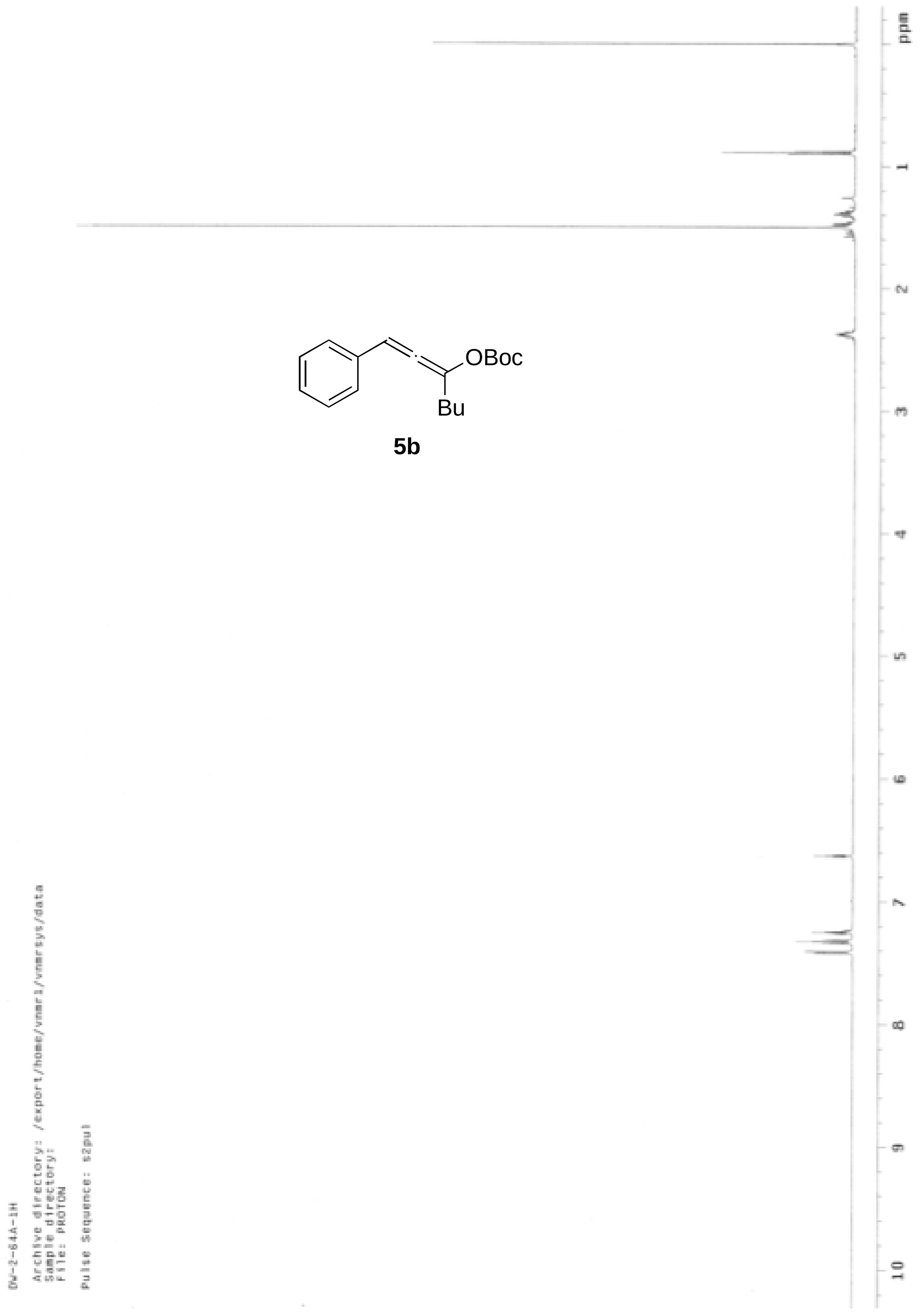
OBoc
Bu
5b
DW-2-64A-1H
Archive directory: /export/home/vnmr1/vnmrsys/data
Sample directory:
File: PROTON
Pulse Sequence: s2pul
ppm
1
2
3
4
5
6
7
8
9
10

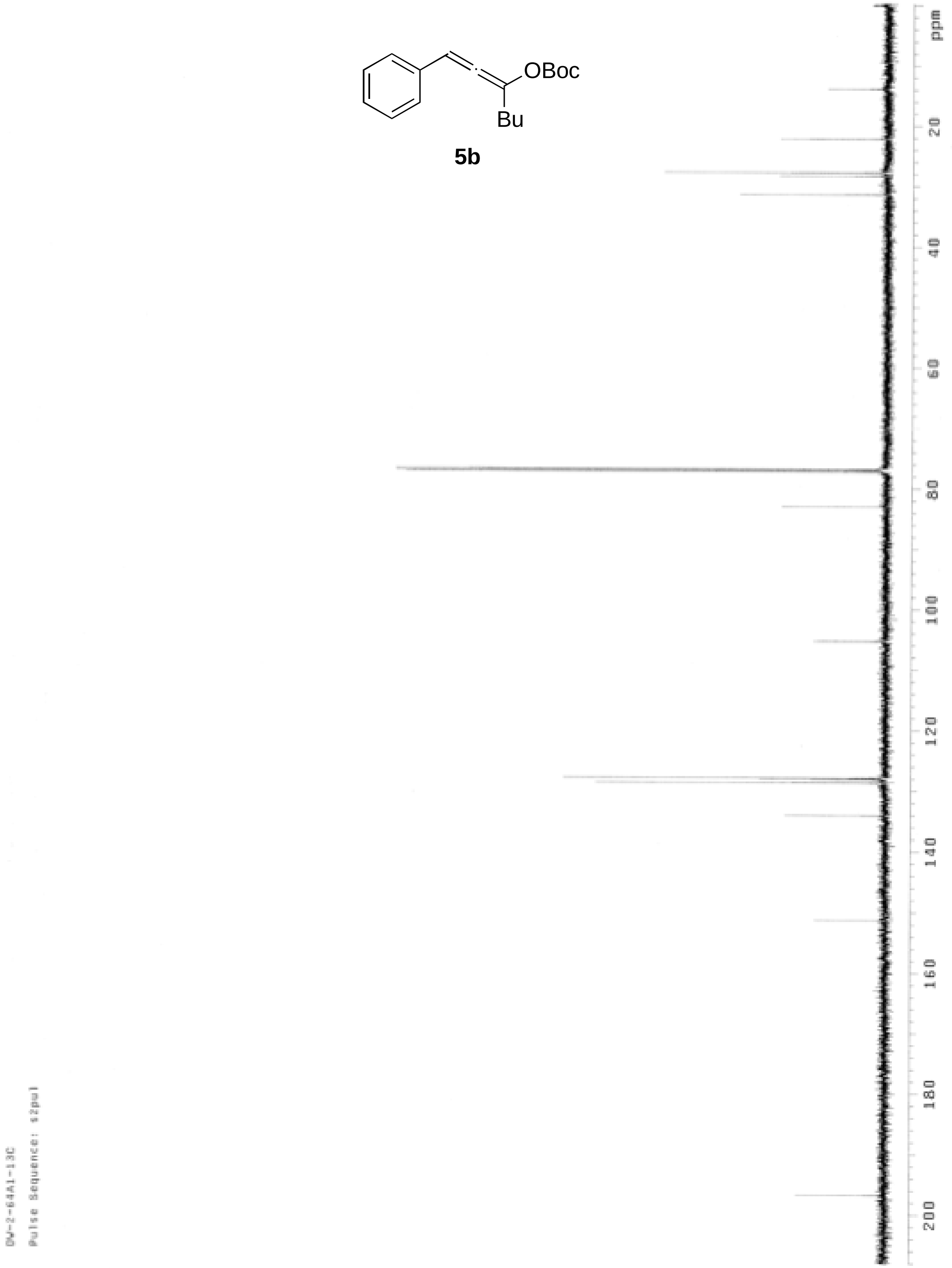

OBoc
Bu
DW-2-64A1-13C
Pulse Sequence: s2pul
ppm
20
40
60
80
100
120
140
160
180
200

5b

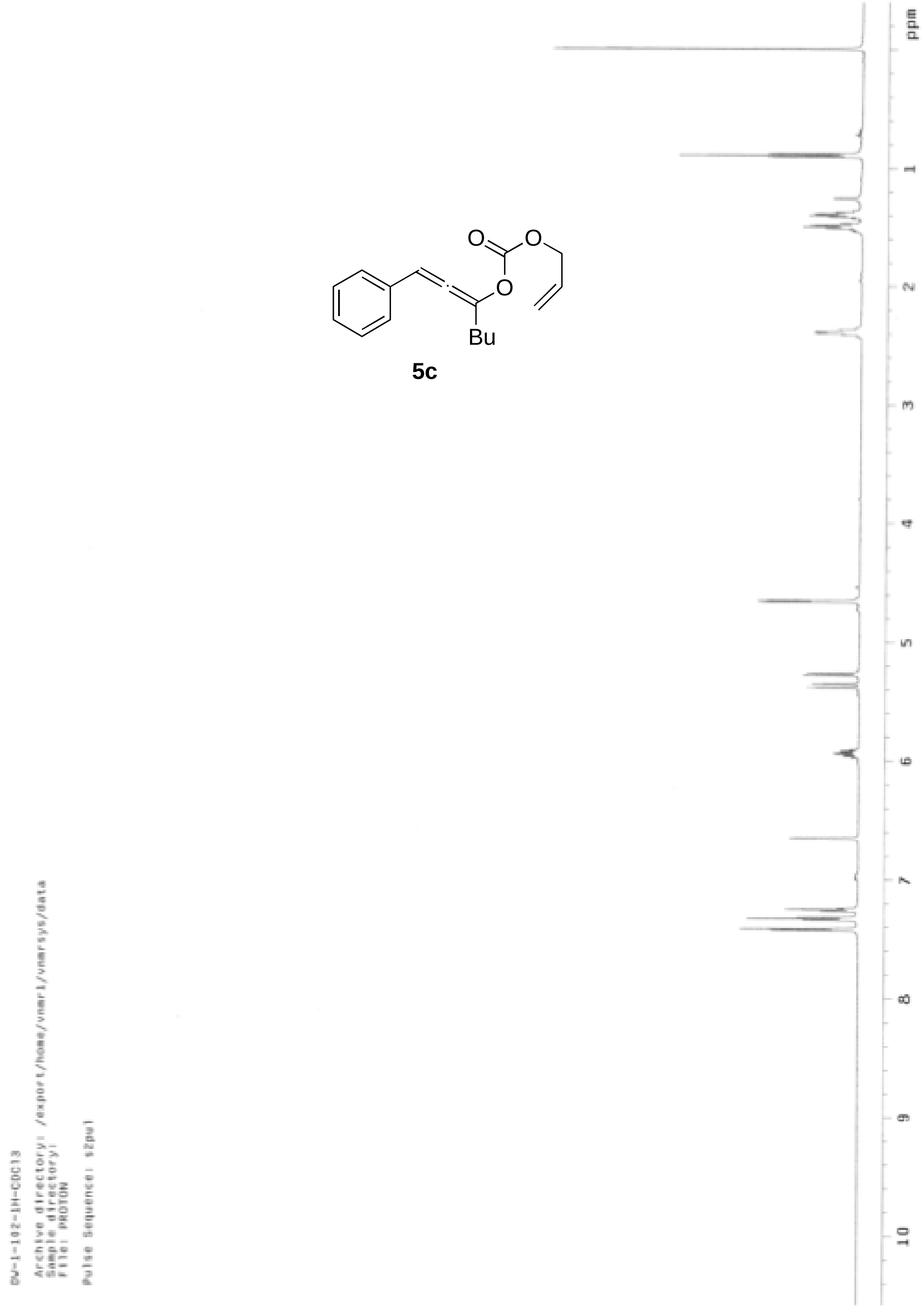
O
O
O
Bu
DV-I-102-1H-CDCl3
Archive directory: /export/home/vnmr1/vnmrsys/data
Sample directory:
File: PROTON
Pulse Sequence: s2pul
ppm
1
2
3
4
5
6
7
8
9
10

5c

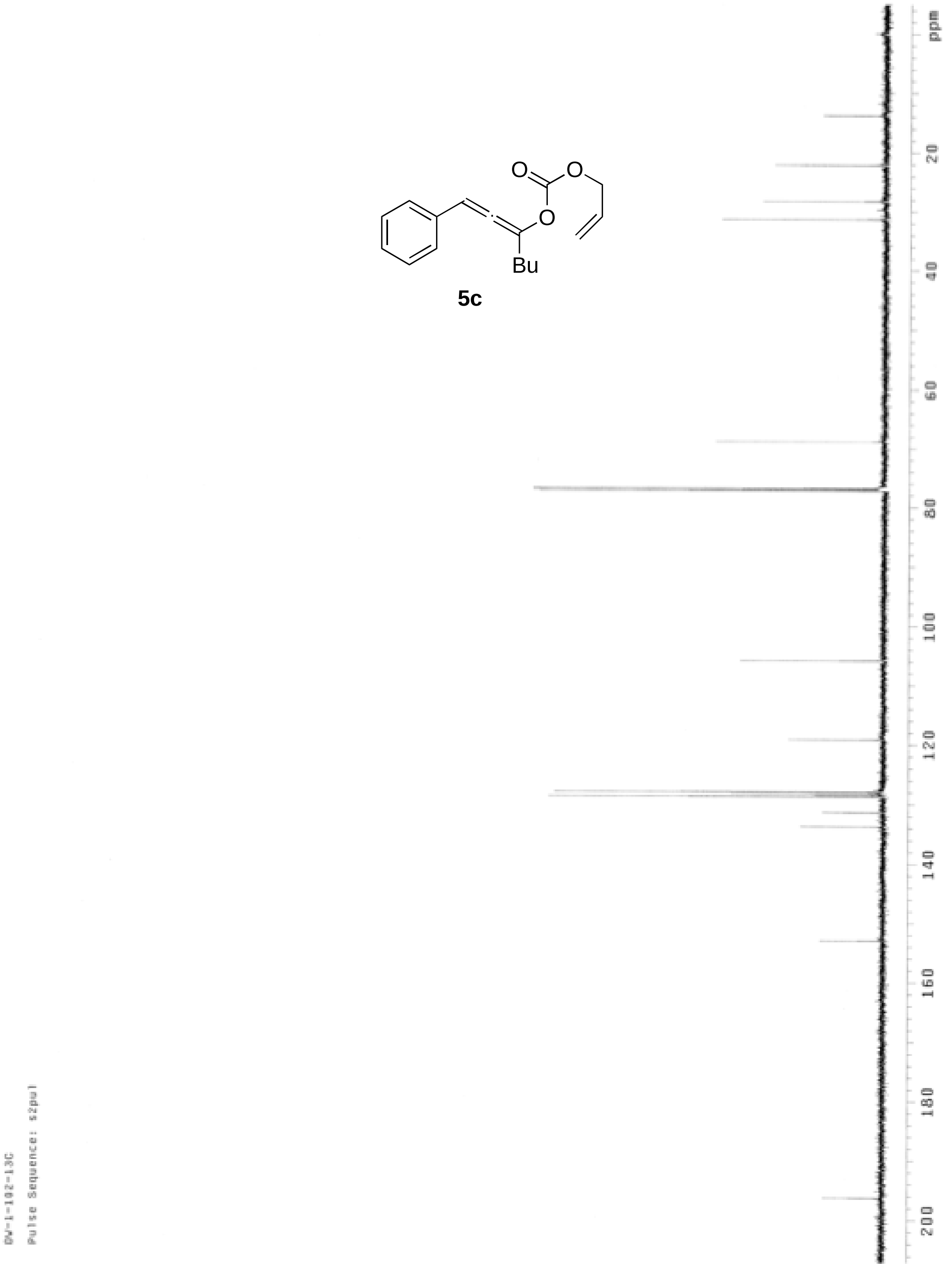
O
O
O
Bu
DW-1-102-13C
Pulse Sequence: s2pul
ppm
20
40
60
80
100
120
140
160
180
200

5c

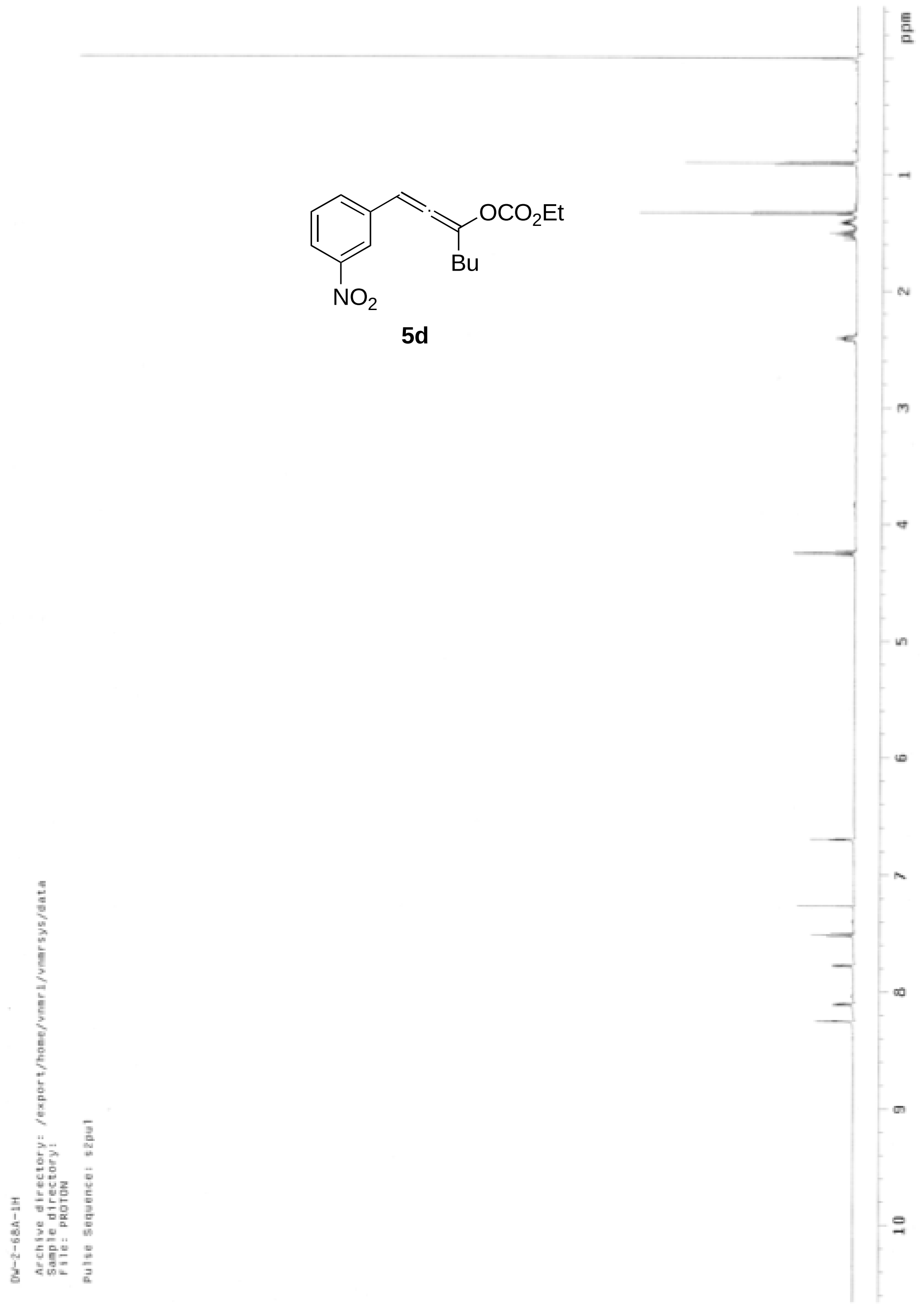
OCO2Et
Bu
NO2
5d
DW-2-68A-1H
Archive directory: /export/home/vnmr1/vnmrsys/data
Sample directory:
File: PROTON
Pulse Sequence: s2pul
ppm
1
2
3
4
5
6
7
8
9
10

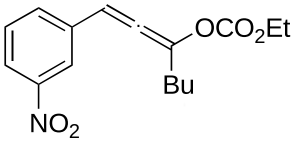

5d

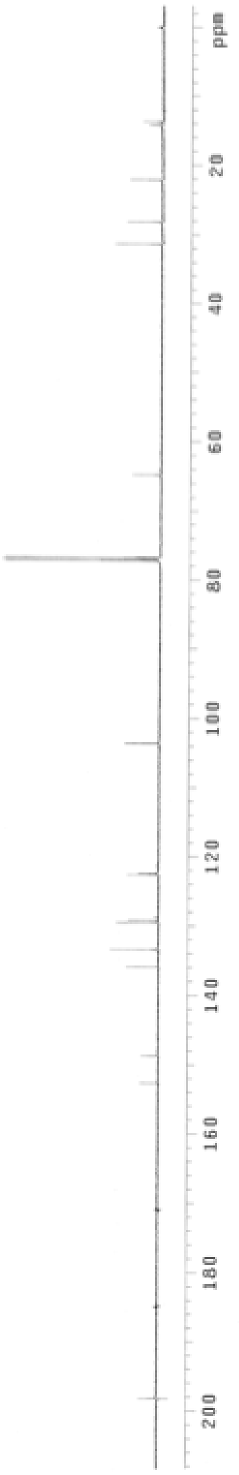

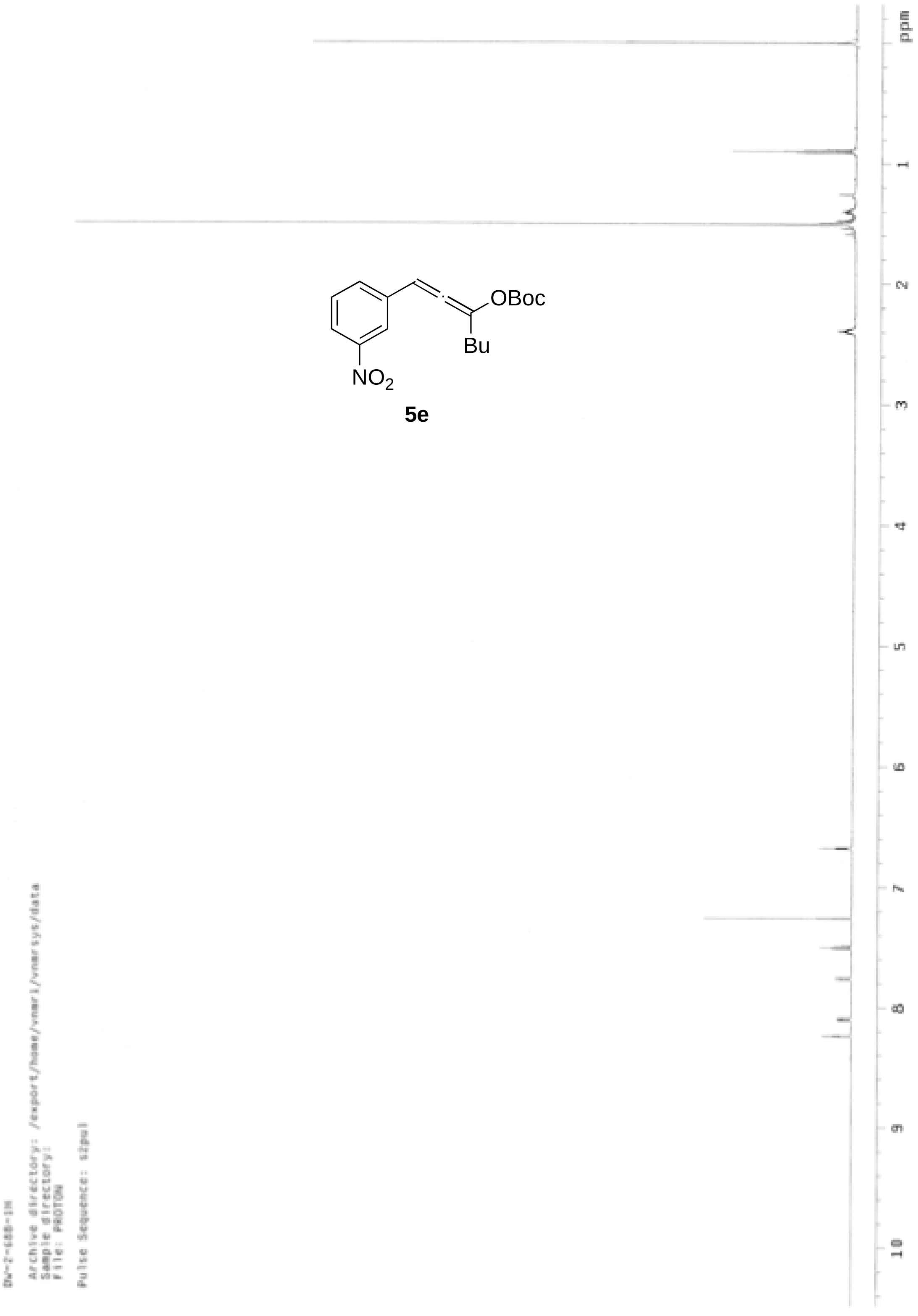

5e

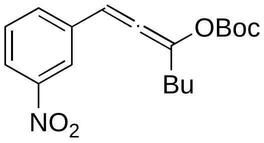

5e

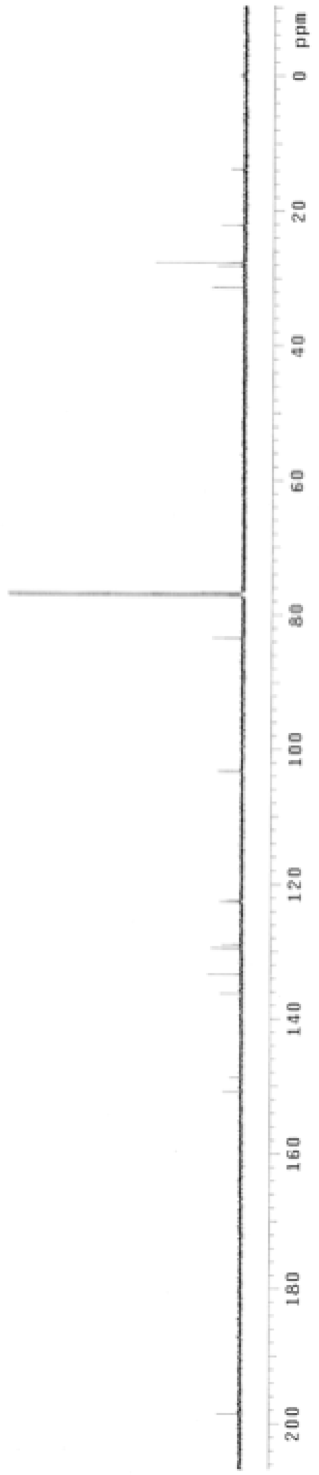

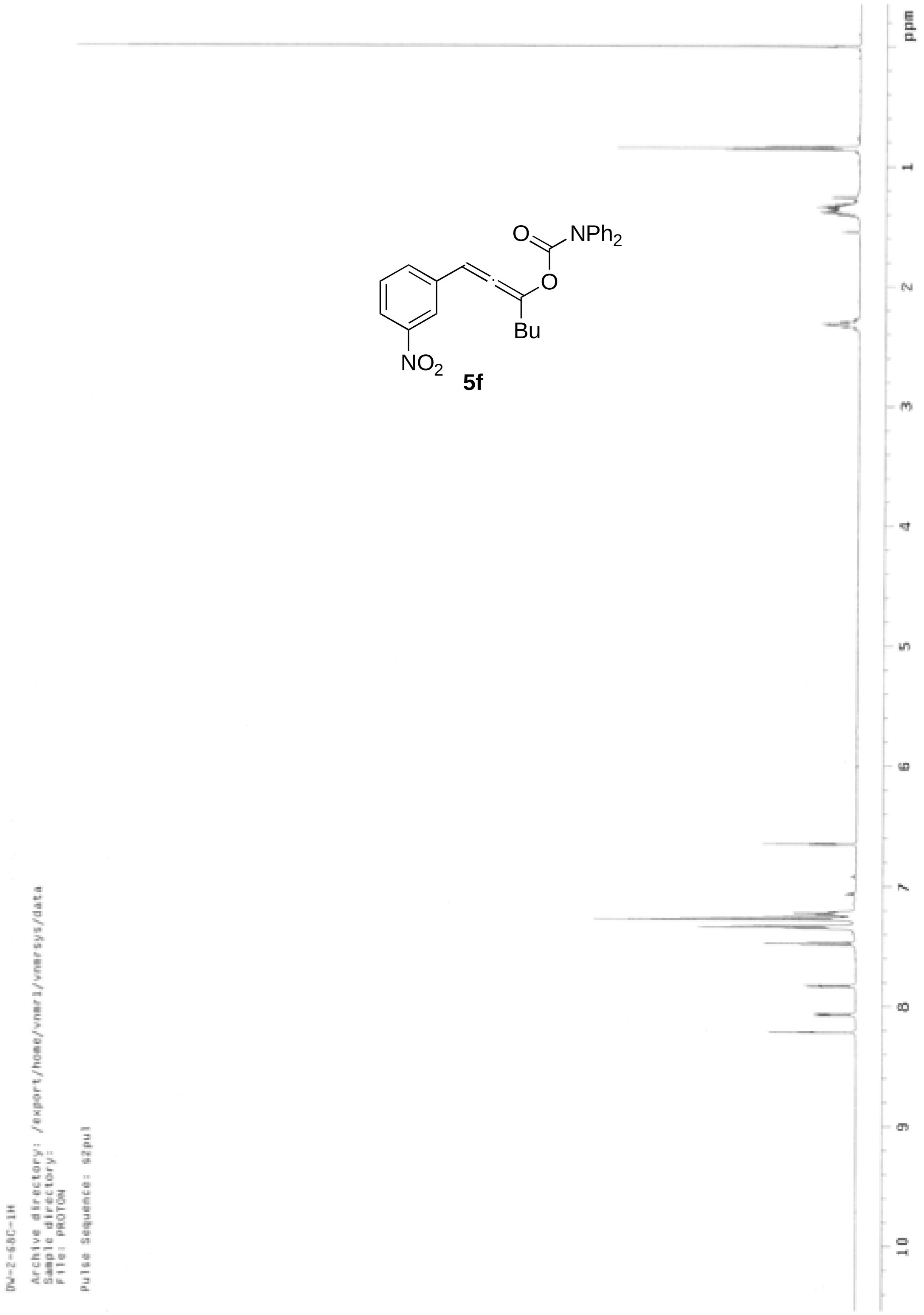
O
NPh2
O
Bu
NO2
5f
DW-2-68C-1H
Archive directory: /export/home/vnmr1/vnmrsys/data
Sample directory:
File: PROTON
Pulse Sequence: s2pul
ppm

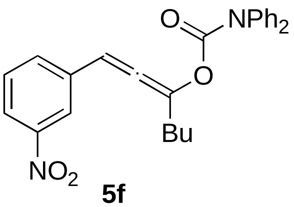
O
NPh2
O
Bu
NO2

5f

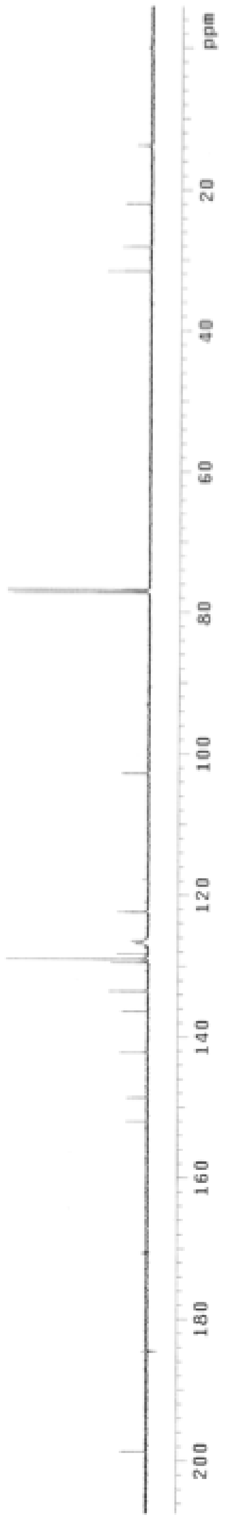
DW-2-68C-13C
Pulse Sequence: s2pul
ppm
20
40
60
80
100
120
140
160
180
200
